# TP63 basal cells are indispensable during endoderm differentiation into proximal airway cells on acellular lung scaffolds

**DOI:** 10.1038/s41536-021-00124-4

**Published:** 2021-03-05

**Authors:** Claudia Bilodeau, Sharareh Shojaie, Olivia Goltsis, Jinxia Wang, Daochun Luo, Cameron Ackerley, Ian M Rogers, Brian Cox, Martin Post

**Affiliations:** 1 Translational Medicine Program, Peter Gilgan Centre for Research and Learning, The Hospital for Sick Children, Toronto Ontario, M5G1X8 Canada; 2 Department of Laboratory Medicine and Pathobiology, University of Toronto, Toronto Ontario, M5S 1A8 Canada; 3 Department of Physiology, University of Toronto, Toronto Ontario, M5S 1A8 Canada; 4 Institute of Medical Sciences, University of Toronto, Toronto Ontario, M5S 1A8 Canada; 5 Lunenfeld-Tanenbaum Research Institute, Sinai Health System, Toronto Ontario, M5T 3H7 Canada

**Keywords:** Embryonic stem cells, Tissue engineering, Stem-cell differentiation

## Abstract

The use of decellularized whole-organ scaffolds for bioengineering of organs is a promising avenue to circumvent the shortage of donor organs for transplantation. However, recellularization of acellular scaffolds from multicellular organs like the lung with a variety of different cell types remains a challenge. Multipotent cells could be an ideal cell source for recellularization. Here we investigated the hierarchical differentiation process of multipotent ES-derived endoderm cells into proximal airway epithelial cells on acellular lung scaffolds. The first cells to emerge on the scaffolds were TP63^+^ cells, followed by TP63^+^/KRT5^+^ basal cells, and finally multi-ciliated and secretory airway epithelial cells. TP63^+^/KRT5^+^ basal cells on the scaffolds simultaneously expressed KRT14, like basal cells involved in airway repair after injury. Removal of TP63 by CRISPR/Cas9 in the ES cells halted basal and airway cell differentiation on the scaffolds. These findings suggest that differentiation of ES-derived endoderm cells into airway cells on decellularized lung scaffolds proceeds *via* TP63^+^ basal cell progenitors and tracks a regenerative repair pathway. Understanding the process of differentiation is key for choosing the cell source for repopulation of a decellularized organ scaffold. Our data support the use of airway basal cells for repopulating the airway side of an acellular lung scaffold.

## Introduction

Extracellular matrix (ECM) is a complex three-dimensional network of proteins that is crucial for cell attachment, proliferation, and differentiation^1–3^. ECM is organ-specific and its composition and mechanical properties varies across organs^2,4^. This complexity has limited the successful artificial reproduction of native ECMs for organ transplants^5^. Whole-organ decellularization is a process that preserves the specific microarchitecture and mechanical properties of the ECM specific to each organ^2,4,6–8^. Decellularized whole-organ scaffolds have been used for organ bioengineering and represent a promising approach to palliate the donor organ shortage for transplantation^2,9–11^. When properly matched, utilization of decellularized scaffolds for transplantation reduces the immunological response and prevents the need for immunosuppressants^12,13^. An important step in organ engineering is the choice of cells to repopulate the acellular organ scaffold^13^. Recellularizing an acellular scaffold of a complex organ like the lung requires multiple cell types^14^. Hence, reseeding with a single multipotent cell population is attractive as the scaffold ECM may guide the differentiation of these cells to the various cell types of the lung. Previously, we have reported that definitive endoderm (DE) cells, derived from embryonic stem cells or induced pluripotent cells, seeded and cultured on rat acellular lung scaffolds in serum- and growth factor-free media differentiate into proximal airway epithelial cells^8^. Specification to proximal airway cells appears to depend on heparan sulfate proteoglycans and bound molecules remaining on the scaffold^8^. The hierarchical differentiation process of endoderm cells to airway cells on the decellularized lung scaffold, however, remains to be elucidated. Such information will be crucial for optimizing the repopulation of the scaffold for lung replacement therapy.

Differentiation of multipotent DE cells on denuded lung ECM scaffolds may in part replicate airway repair. Basal cells are considered to be the intrinsic stem cell of the airway epithelium as they function as their progenitor during repair^15^. Basal cells account for ~30% of the pseudostratified columnar airway epithelium^15,16^. They express the transcription factor TP63 (Tumor protein p63), a homolog of the tumor suppressor protein p53^17^. TP63 has been shown to regulate the epithelial differentiation program, especially in stratified epithelia such as skin, prostate, cornea, and breast^18–21^. Using alternative promoters, the *p63* gene generates transcripts encoding TAp63 and ΔNp63 isoforms^17^. In the lung, ΔNp63 is the predominant isoform and its expression is restricted to basal cells of the tracheobronchial epithelium^22,23^. Basal cells in the airways are further characterised by the expression of keratins 5 (KTR5) and 14 (KRT14) in conjunction with TP63^16,24^. Endodermal TP63^+^ cells are already present at the onset (E9.5) of lung development^25,26^. They can give rise to proximal and alveolar lineages although the capability to alveolar lineages is lost at E10.5^26^. TP63^+^ cells that will become basal cells in the adult lung arise around E13.5-14.5 prior to any expression of KRT5 and 14^26^. These TP63^+^ basal cells start to co-express KRT5 and 14 at birth^24^. In normal healthy state, most mature basal cells in the lung express KRT5 while only a few express KRT14^27^. However, after airway injury, expression of KRT14 increases, specifically during the repair process^27,28^. Knockouts of *Tp63* [29, 30] and the *ΔNp63* isoform^29^ in mice have revealed a critical role of TP63 in the maintenance of progenitor populations that encourage epithelial development and morphogenesis, although TP63 appears dispensable for lineage commitment and differentiation^30^. In the lung, loss of TP63 results in airways being lined with a simple epithelium that lack basal cells^20^. Airway cells deficient in TP63 are unable to maintain their integrity and to form a pseudostratified epithelium^23^.

Here we demonstrate that TP63^+^ epithelial cells arise during early lung specification of definitive endoderm cells on acellular lung scaffolds. These multipotent TP63^+^ cells then give rise to ciliated, secretory and mature basal cells making up a pseudostratified columnar airway epithelium that is abrogated by removal of TP63.

## Results

### Differentiation of DE cells on acellular lung scaffolds resembles airway epithelium development

**Fig. 1:**
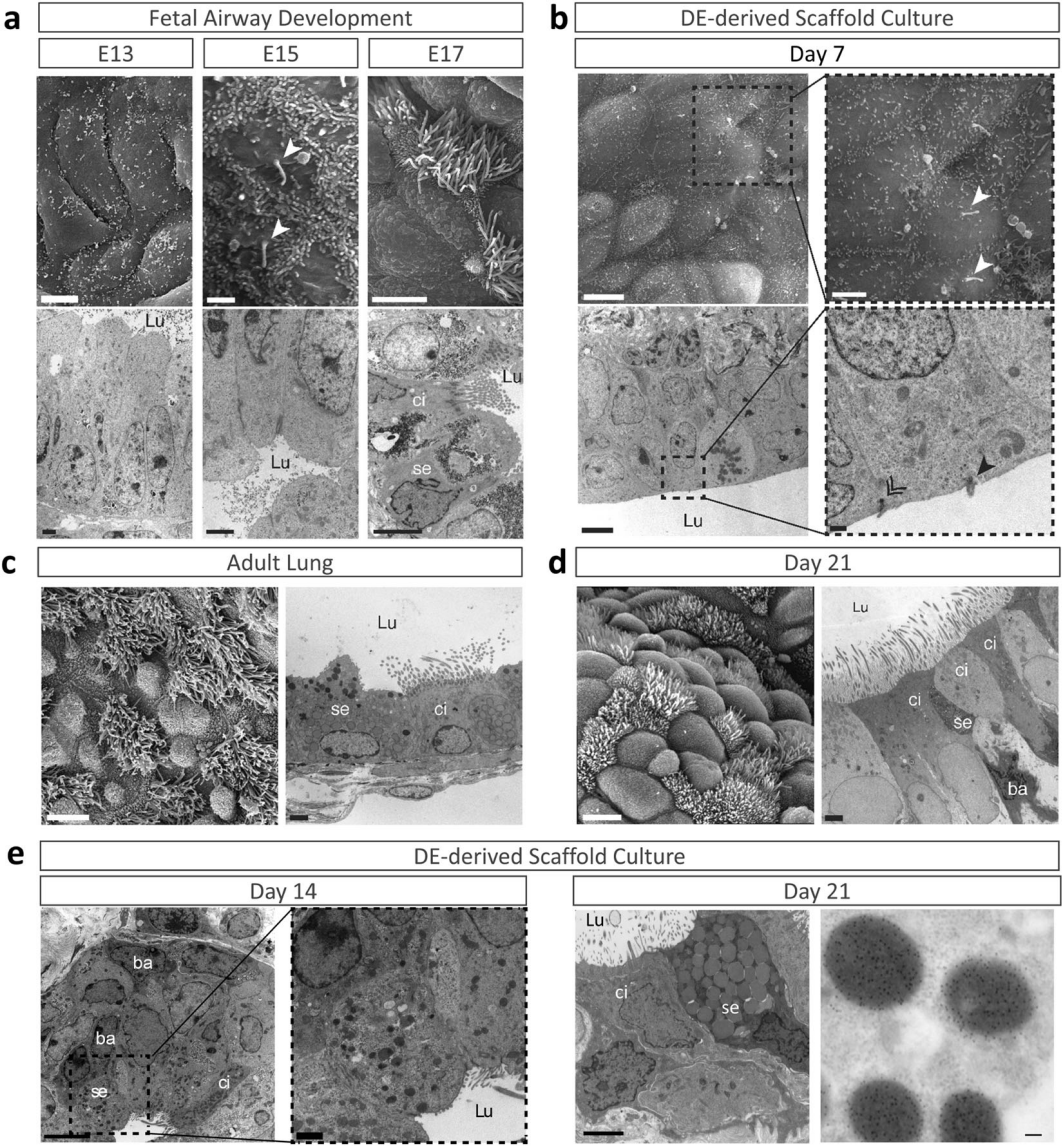
Differentiation of definitive endoderm (DE) cells on decellularized lung scaffolds resembles in situ epithelial differentiation of native mouse airways. **a** Progression of airway development in murine lung visualized by SEM (top panel) and TEM (bottom panel). **b** Electron micrographs of DE-scaffolds at day 7 of culture. **c** Electron micrographs of adult native airway epithelia. **d** Electron micrographs of DE-scaffolds at day 21 of culture. **e-**left panel Electron micrographs and immuno-TEM of DE-scaffolds at days 14 of culture. **e**-right-panel Immunogold labelling for SCGB1A1 of club cells at day 21. SEM, scanning electron microscopy; TEM, transmission electron microscopy; Lu, lumen; ci, ciliated cell; se, secretory cell; ba, basal cell. Scale Bars: **a** SEM 5 μm, TEM 2 μm; **b** SEM-left 5 μm, TEM-left 2 μm, SEM-right 1 μm; TEM-right 500 nm; **c**, **d** SEM 5 μm, TEM 2 μm; **e** TEM 2 µm, Immuno TEM-right 250 nm.

Previously, we demonstrated that ES-derived definitive endoderm (DE) cells differentiated into proximal airway epithelial cells when seeded on acellular lung scaffolds under serum- and growth factor-free conditions^8^. To better understand the hierarchical differentiation pattern in vitro, we compared the differentiation of murine DE cells on acellular lung scaffolds to the development of airway epithelium in mice using transmission (TEM) and scanning (SEM) electron microscopy (Fig. 1). After 7 days of culture on the scaffolds, monociliated cells appear that resemble the monociliated pseudostratified epithelial cells lining the airways of E13-15 mouse lung (Fig. 1b, Supplementary Fig. 1). In situ, monociliated epithelial cells disappear when pseudostratified multiciliated columnar epithelial cells emerge at E17 (Fig. 1a) while, in vitro, multiciliated epithelial cells appear at day 14 of culture (Supplementary Fig. 1). Occasionally, secretory cells are visible at day 14 of culture (Fig. 1e). At day 21 of culture, differentiated DE cells on the scaffolds have reorganized into airway epithelial structures that architecturally look like native mouse airway epithelium, with the presence of ciliated, secretory, and basal cells (Fig. 1c, d). Fully differentiated club cells with granules filled with secretory protein SCGB1A1 are frequently detected (Fig. 1e). Combining these ultrastructural observations with immunostaining for basal (TP63, KRT5), club (SCGB1A1) and ciliated (TUBB4A) lineage markers revealed rapid differentiation into TP63^+^ and KRT5^+^ basal cells, i.e., within 4 to 7 days after seeding of DE cells onto the scaffolds, respectively (Supplementary Fig. 1). In agreement with the EM findings, TUBB4A^+^ (ciliated) airway cells were detected at day 14 of culture while SCGB1A1^+^ (club) cells were spotted at day 21 (Supplementary Fig. 1). With advancing differentiation TP63^+^/KRT5^+^ basal cells became less abundant and positioned themselves on the basolateral side of the pseudostratified airway epithelium (Supplementary Fig. 1, Supplementary Fig. 3a). Thus, differentiation of endoderm cells on acellular lung scaffolds into airway epithelial cells recapitulate some aspects of natural development of airway epithelium with TP63^+^ basal cells being one of the first identifiable airway cells^25,31^.

### Early differentiation of DE cells into basal cells on lung acellular scaffolds

**Fig. 2:**
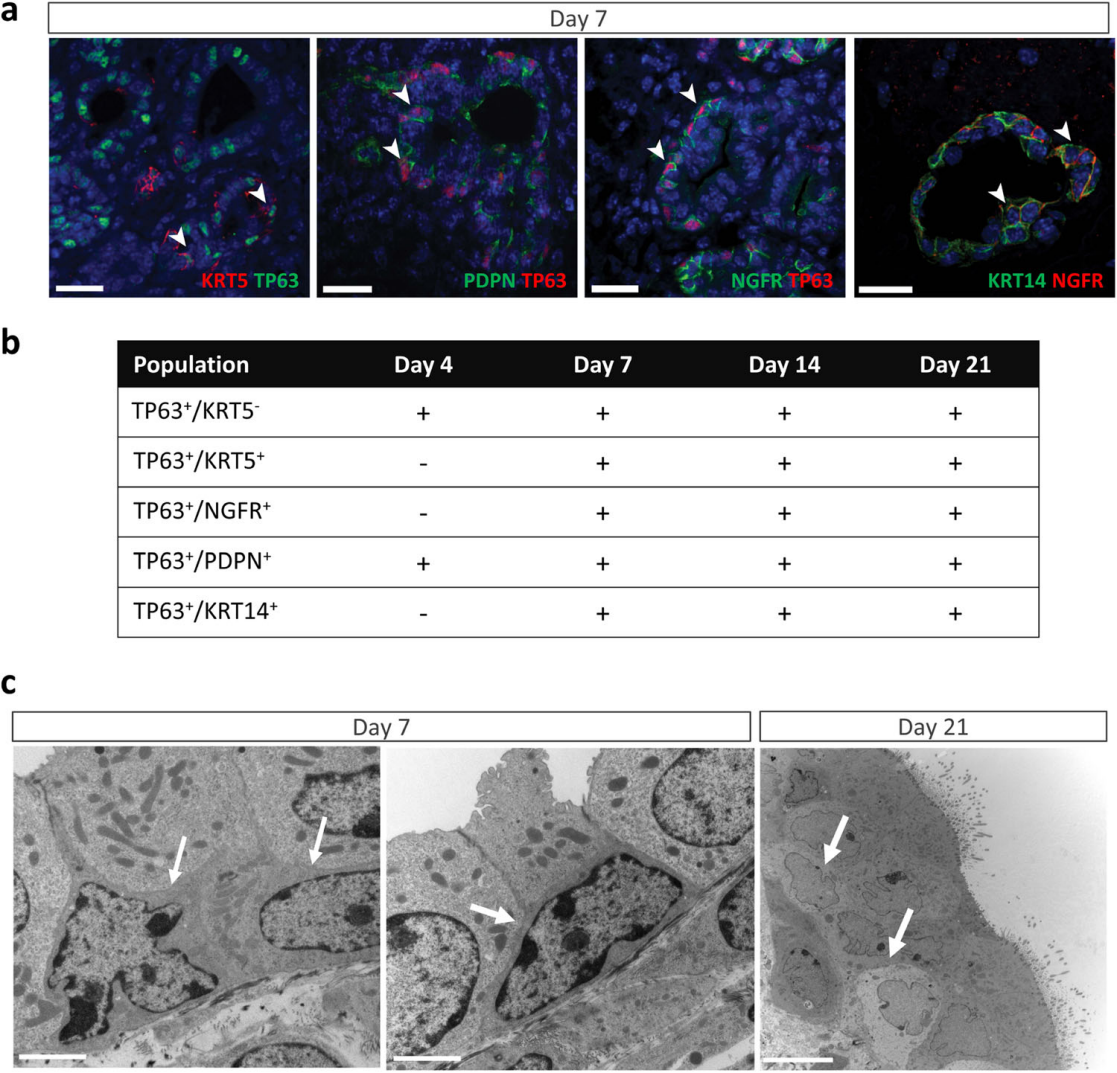
Identification of basal cells on decellularized lung scaffolds seeded with definitive endoderm (DE) cells. **a** Representative immunofluorescence confocal images of DE cells on acellular lung scaffolds after 7 days of culture co-stained for a combination of basal lineage cell markers (arrow heads) including TP63 (tumor protein p63), KRT5 (keratin 5), KRT14 (keratin 14), PNDP (podoplanin) and NGFR (nerve growth factor receptor). Scale bar: 25 μm. **b** Table summarizing different combinations of basal cell markers detected between 4 and 21 days of culture. “+”: detected, “−”: not detected. **c** Representative TEM images of basal cells (arrow) at day 7 and 21 of culture of DE cells on acellular lung scaffolds. Scale bar: 2 μm (Day 7), 10 μm (Day 21).

To explore the early differentiation of DE cells into basal cells on acellular lung scaffolds, cell-scaffold cultures at various time points were stained for a panel of well-known basal cell markers: TP63, KRT5, KRT14, PDPN, and NGFR^15,24,32–34^. TP63-positive cells were already present after 4 days of seeding the scaffolds with DE cells (Supplementary Fig. 1, Fig. 2a, b, Supplementary Fig. 3a). Some TP63^+^ cells co-expressed PDPN; however, canonical lineage markers KRT5 and 14 were absent (Fig. 2b), suggesting that the TP63^+^ cells at day 4 of culture are mostly immature basal cells^24,25^. After 7 days of culture, various positive combinations of TP63 with other basal cell markers emerged (Fig. 2a, b). The basal cell populations were heterogeneous (Fig. 2b), with some expressing only TP63, while others being positive for both TP63 and KRT5 (Supplementary Fig. 2a). A rarer population of TP63^-^/KRT5^+^ cells was also identified on the scaffolds (Supplementary Fig. 2a). The presence of basal cells at day 7 of culture was confirmed at the ultrastructural level using TEM (Fig. 2c) and their cell identity was corroborated by positive immunogold labeling for TP63 and KRT5 (Supplementary Fig. 2b). Early and swift differentiation of DE cells into basal cells between days 4 and 7 of culture was substantiated by increased gene expression of *Tp63, Krt5*, and *Krt14* (Supplementary Fig. 3b). *Tp63* gene expression remained high compared to freshly sorted endoderm cells but dropped gradually with longer culture durations, in line with the decrease in positive staining for TP63^+^/KRT5^+^ (Supplementary Fig. 3a). Most epithelial cells lining the tubule-like structures on the scaffolds at day 21 expressed the proximal lung lineage marker SOX2 with the underlying basal cells being immune-positive for SOX2 and TP63 (Supplementary Fig. 3c). Developmentally TP63^+^ cells contribute to all proximal lineages^26^ while TP63^+^ basal cells in the adult lung act as local stem cells to regenerate airway epithelium after injury^15,28,35^. Thus, their early appearance prior to any other identifiable airway cell type on the scaffolds fits with the concept of them being progenitor cells for proximal airway cells.

### TP63-deficient DE cells do not differentiate into proximal airway cells on acellular lung scaffolds

**Fig. 3:**
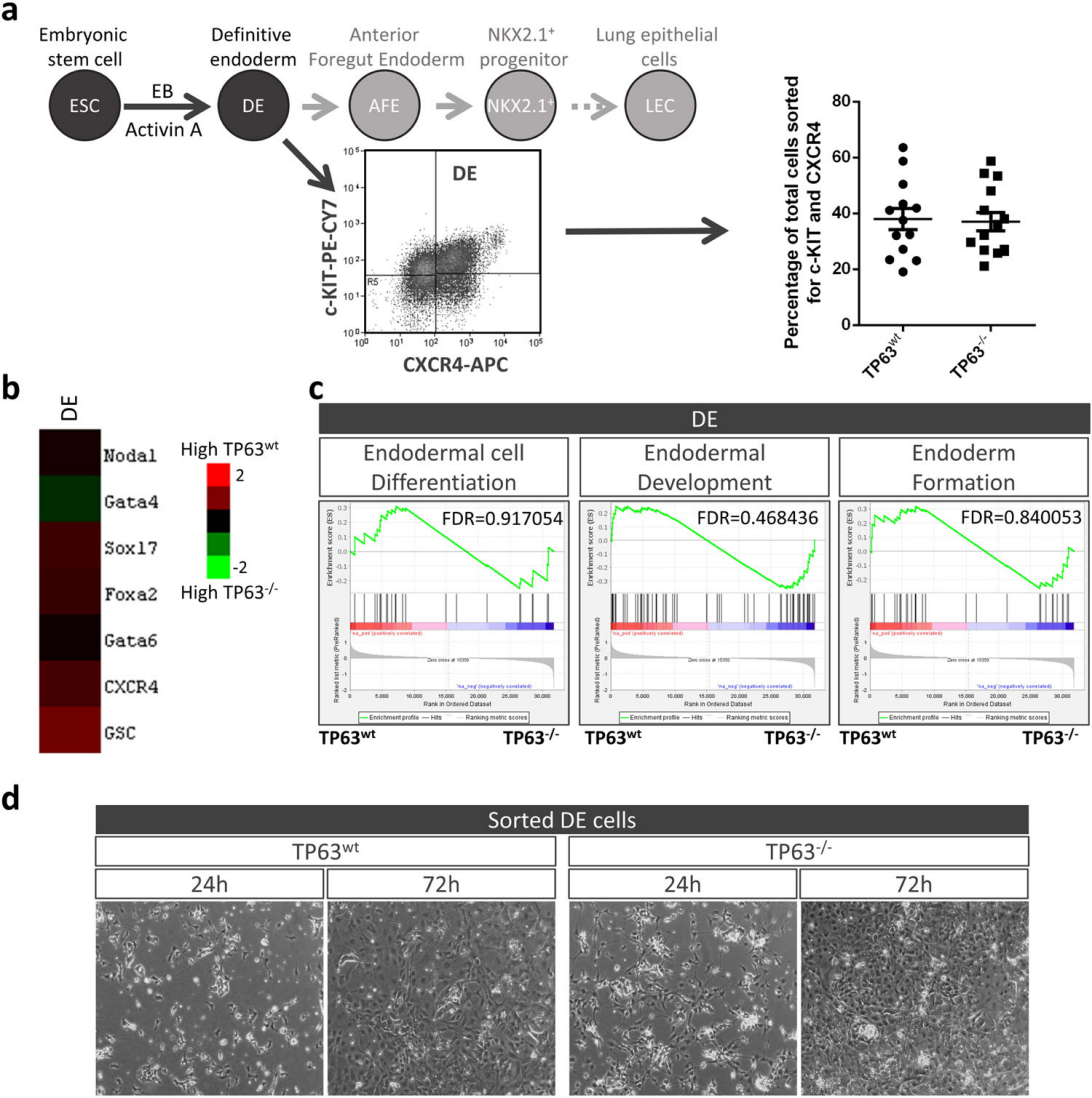
TP63-deficient embryonic stem cells differentiate into definitive endoderm. **a-**upper panel Schematic of ES cell differentiation into DE and lung progenitor cells. **a-**bottom panel Potential of TP63-deficient ES cells to differentiate into double-positive CXCR4/c-KIT DE cells (mean ± SEM, *n* ≥ 9 separate differentiations). **b** Heat map of endoderm-associated genes shows no difference in expression between freshly sorted wildtype and TP63-deficient DE cells. Measured by RNA-seq of CXCR4^+^/c-KIT^+^ sorted DE cells, *n* = 3 separate DE sorts. **c** GSEA confirms similarity (FDR > 0.1) of endoderm-associated gene expression between wildtype and TP63-deficient DE cells. **d** Attachment and proliferation of CXCR4^+^/c-KIT^+^ sorted wildtype and TP63^−/−^ DE cells plated on gelatin in SFDM without growth factors (representative of *n* = 3 separate DE sorts).

To determine whether TP63^+^ cells are an early progenitor population emerging from endoderm cells seeded on acellular lung scaffolds, we created TP63-deficient ES cells using CRISPR/Cas9 (Supplementary Fig. 4a). Insertion of mCherry and a puromycin selection marker in one allele led to a deletion of 4 nucleotides in the other allele of *Tp63*. After cloning and verifying the mutation, we first confirmed that the TP63-deficient ES cells could differentiate into the three germ layers (Supplementary Fig. 4b). After spontaneous embryonic body differentiation, endoderm was visualized with anti-FOXA2, mesoderm with anti-SMA, and ectoderm with anti-TUBB3. No obvious changes in differentiation potential toward the three germ layers between TP63-deficient (TP63^−/^^−^) and wildtype (TP63^wt^) ES cells were noted. The efficiency to differentiate into definitive endoderm cells was also unchanged. The percentage of TP63^−/−^ cells co-expressing the surface markers c-KIT and CXCR4 after embryonic body formation and exposure to 50 ng/ml activin A was similar to wildtype cells (Fig. 3a). Transcriptome analysis also revealed no differences in gene expression of DE-associated genes between c-KIT^+^/CXCR4^+^ sorted TP63^−/−^ and TP63^wt^ endoderm cells (Fig. 3b, c). To ensure viability after sorting, TP63^−/−^ DE cells were seeded on gelatin-coated plates in serum-free differentiation medium (SFDM); the same medium used in the scaffold experiments. TP63-deficient DE cells exhibited similar attachment and proliferation properties as TP63^wt^ DE cells (Fig. 3d).

**Fig. 4:**
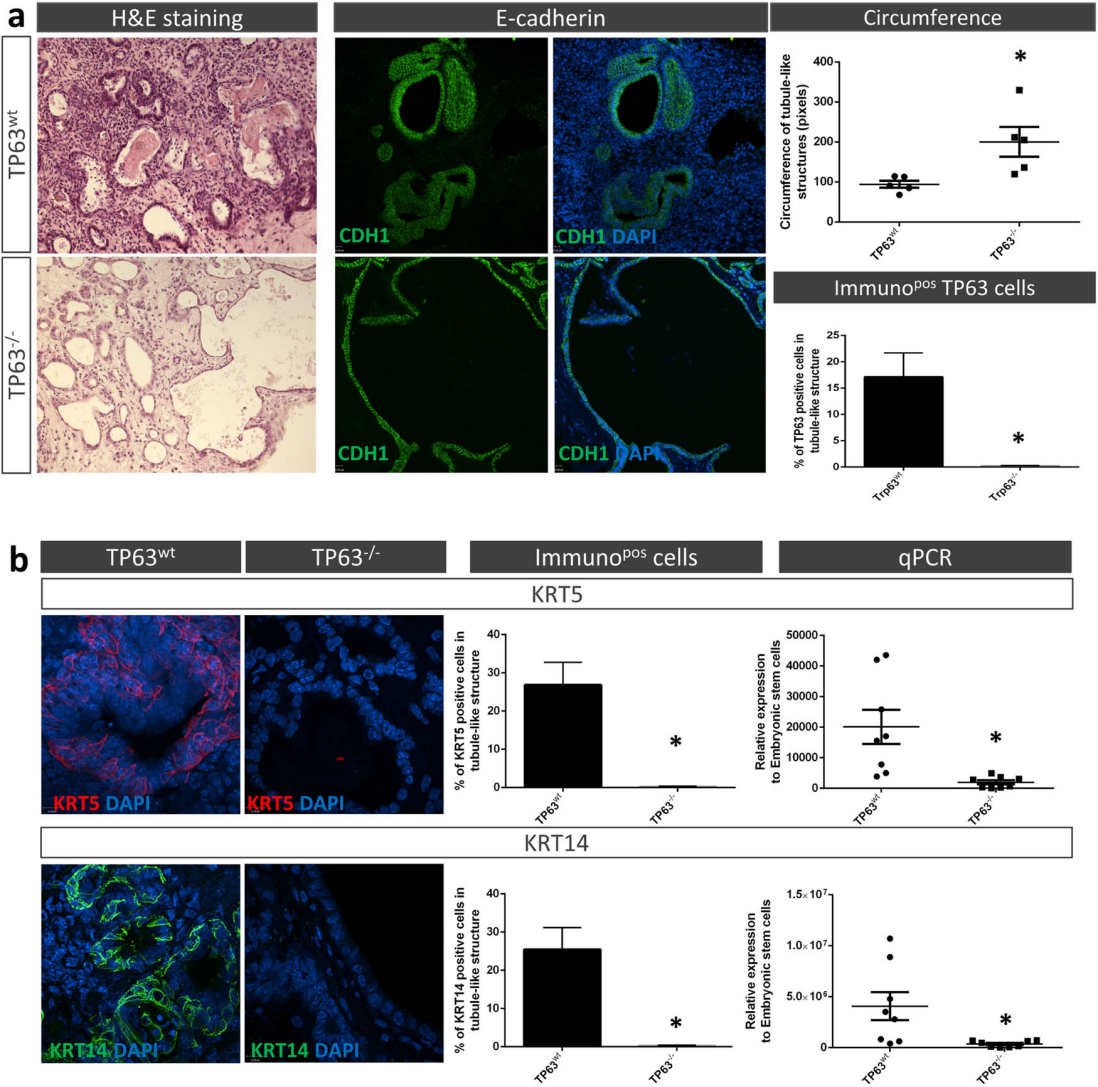
TP63-deficient definitive endoderm (DE) cells seeded on decellularized lung scaffolds do not differentiate into basal cells. **a** DE cells seeded on acellular lung scaffolds after 21 days of culture form tubule-like structures (left panels) that are lined with CDH1^+^ epithelial cells (middle panels). Loss of TP63 leads to larger tubule-like structures (a-top right panel) devoid of TP63^+^ cells (bottom right panel). Data are expressed as mean ± SEM, *n* ≥ 12 different scaffolds, *p < 0.05. **b**-left panel Representative immunofluorescence confocal images and quantification of KRT5^+^ and KRT14^+^ epithelial cells in tubule-like structures of wildtype and TP63^−/−^ DE cells seeded on acellular lung scaffolds (mean ± SEM, *n* ≥ 16 different scaffolds, *p < 0.05). **b**-right panel Quantitative PCR of *Krt5* and *Krt14* gene expression in wildtype and TP63^−/−^ DE cells seeded on acellular lung scaffolds (mean ± SEM, *n* = 8 separate scaffold cultures, *p < 0.05).

**Fig. 5:**
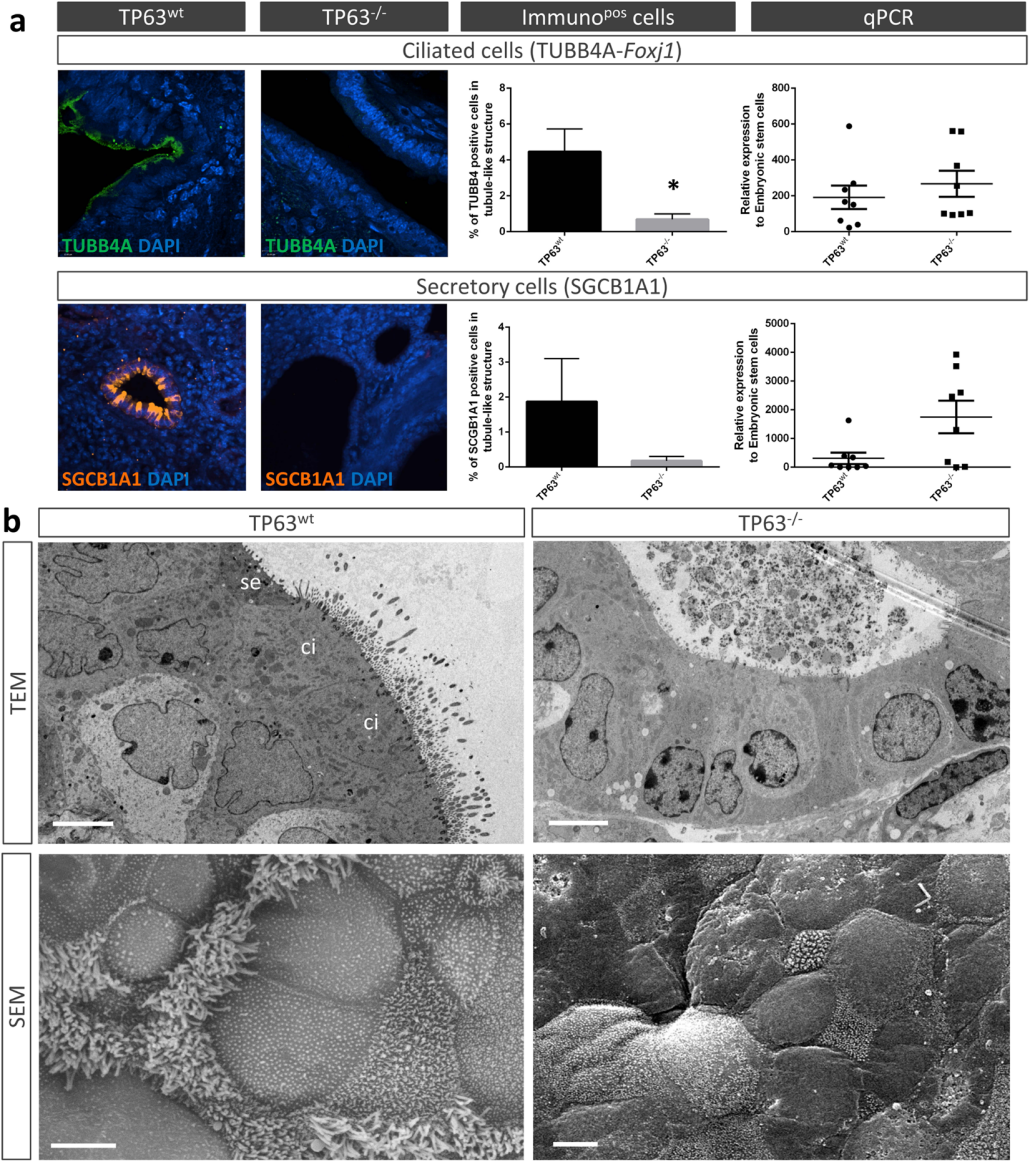
TP63-deficient definitive endoderm (DE) cells seeded on decellularized lung scaffolds do not differentiate into proximal airway cells. **a-**left panel Representative immunofluorescence confocal images and quantification of TUBB4A^+^ and SCGB1A1^+^ epithelial cells in tubule-like structures after 21 days of culture (mean ± SEM, *n* ≥ 16 different scaffolds, *p < 0.05). **a**-right panel Quantitative PCR of *Foxj1* and *Scgb1a1* gene expression in wildtype *vs* TP63^−/−^ DE cell-scaffold cultures (mean ± SEM, *n* = 8 separate scaffold cultures). **b** Representative TEM and SEM images of wildtype *vs* TP63^−/−^ DE cell-scaffolds after 21 days of culture (*n* = 3 separate scaffold cultures). se, secretory cell; ci, ciliated cell. Scale bar: 5 μm.

Sorted TP63^−/−^ and TP63^wt^ endoderm cells were then seeded on acellular lung scaffolds and cultured for 21 days in SFDM media only. Histological analysis at day 21 identified numerous tubule-like structures lined with CDH1^+^ epithelial cells on the scaffolds independent of the presence or absence of TP63 (Fig. 4a-left panel). However, the tubule-like structures on scaffolds seeded with TP63^−/−^ endoderm cells had significantly larger lumens (>2-fold based on circumference measurements) than the structures on scaffolds seeded with TP63^wt^ endodermal cells (Fig. 4a-right top panel). To determine the presence of basal and proximal airway cells within the tubule-like epithelial structures at day 21, we performed confocal immunofluorescence and gene expression analyses. As anticipated, no TP63^+^ cells were detected within the tubule-like structures of scaffolds seeded with endoderm cells lacking TP63 (Fig. 4a-right bottom panel). Scaffolds also did not contain any TP63-positive cells at day 7 of culture (not shown). In agreement with the absence of TP63^+^ cells, tubule-like structures of scaffolds seeded with TP63^−/−^ endoderm cells stained negative for KRT5 and 14 (Fig. 4b). Absence of mature basal cells in the scaffolds seeded with TP63^−/−^ endoderm cells was confirmed by negative gene expression of *Krt5* and *Krt14*. Immunofluorescence for TUBB4A and SCGB1A1 revealed markedly less multi-ciliated and secretory cells, respectively, within tubule-like structures of scaffolds seeded with endoderm cells lacking TP63 compared to scaffolds seeded with wildtype endoderm cells (Fig. 5a). Ultrastructural analysis using TEM and SEM corroborated the reduction/absence of multi-ciliated and secretory cells within the tubule-like structures of scaffolds seeded with TP63-deficient endoderm cells (Fig. 5b). Pseudostratified ciliated and secretory columnar epithelial cells were easily recognizable in the tubule-like structures of scaffolds seeded with wildtype endoderm cells (Fig. 5b-left panel). In contrast, tubule-like structures of scaffolds seeded with endoderm cells lacking TP63 were lined with prismatic-shaped epithelial cells having microvilli, but no cilia, and lots of debris in the lumen (Fig. 5b-right panel). These epithelial cells contained mitochondria that were larger in size (perimeter and surface area) but had a similar shape (aspect ratio and circularity) as mitochondria of epithelial cells on scaffolds seeded with wildtype endoderm cells; however, their number was significantly reduced (Supplementary Fig. 5a). Interestingly, gene expression of *Foxj1* (marker for ciliated cells^36^) and *Scgb1a1* did not follow the protein expression of TUBB4A and SCGB1A1 (Fig. 5a). Expression of *Foxj1* remained unchanged while that of *Scgb1a1* trended higher in scaffolds seeded with TP63-deficient versus wildtype endoderm cells.

The absence of TP63 did not shift the differentiation of DE cells on the acellular lung scaffolds from proximal toward distal airway cells. Gene expression of *Nkx2.1* (lung progenitor marker^37^) and *Sox2 (*proximal lung lineage marker^38^), respectively, was not significantly changed compared to scaffold cultures seeded with TP63^wt^ endoderm cells (Supplementary Fig. 5b). Furthermore, at day 21, cells lining the tubule-like structures on the scaffolds seeded with TP63-deficient endoderm cells stained positive for SOX2 (Supplementary Fig. 6a), similar to scaffolds seeded with wildtype endoderm cells (Supplementary Fig. 3c). No positive staining for SOX9 (distal lung marker^39^), HOPX (alveolar progenitor cell marker^40,41^), and pro-surfactant protein C (SFTPC, an alveolar type II cell marker^42,43^) was observed (Supplementary Fig. 6a).

Gene expression analysis of lineage markers for thyroid, neuroectoderm, and liver demonstrated significant increases in *Albumin*, but not *Olig2* and *Pax8*, in TP63^−/−^ versus TP63^wt^ DE seeded scaffolds. *FoxA3* showed a positive trend (*p* < 0.07) towards an increase in expression. Thus, loss of TP63 may shift the differentiation of DE cells on the scaffolds towards the posterior foregut endoderm, but not thyroid epithelium and forebrain (Supplementary Fig. 6b).

**Fig. 6:**
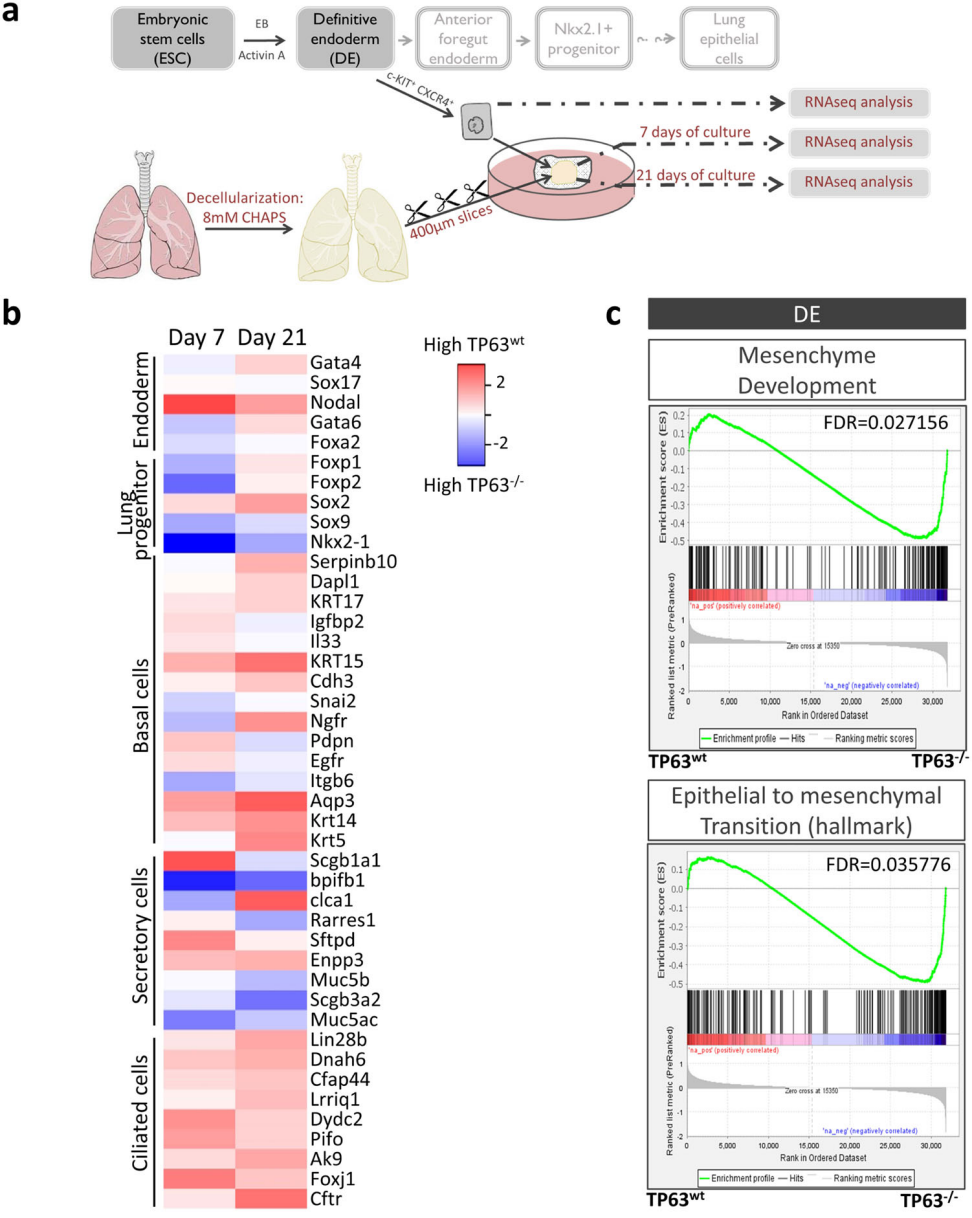
Transcriptome profiling of wildtype and TP63-deficient definitive endoderm (DE) cells seeded on decellularized lung scaffolds. **a** Schematic of RNA-seq experiments. **b** Heat map of genes predominantly associated with endoderm, lung progenitor, basal and proximal (secretory and ciliated) airway cells, respectively; columns show averaged log expression between TP63^wt^ and TRP63^−/−^ DE-scaffolds per time point. **c** GSEA of DE cells for gene sets related to mesenchyme development and epithelial to mesenchymal transition in TP63^−/−^ DE cells compared to wildtype DE cells. RNA sequencing was repeated 4 times using 4 separate wildtype and 3 separate TP63-deficient DE cell-scaffold cultures. Averaged fold change in (**b**) is expressed as log2.

To further explore the differentiation of endoderm cells on the acellular lung scaffolds in the absence of TP63, RNA sequencing (RNA-seq) was performed immediately after DE sorting and after 7 and 21 days of culture of the endoderm cells on the scaffolds (Fig. 6a). The transcriptome analysis corroborated our ultrastructural and immunohistochemical findings and showed that expression of genes associated with basal and ciliated cells, but less of those associated with secretory cells, were markedly reduced in scaffolds seeded with TP63^−/−^ versus TP63^wt^ DE cells (Fig. 6b). Interestingly, gene set enrichment analysis (GSEA) of DE cells directly after sorting revealed that endoderm cells lacking TP63 were significantly enriched in sets of genes related to mesenchyme development and epithelial to mesenchymal transition (Fig. 6c). We identified 933 and 550 differentially expressed genes (DEG) with a false discovery rate (FDR) lower than 0.05 in scaffolds seeded with TP63-deficient versus wildtype DE cells at 7 and 21 days of culture, respectively (Supplementary Table 2). Anatomical term and pathway enrichment (PE) analysis, using Mousemine, were performed to determine the significance of these DEGs. At day 7, we found 59 anatomical terms enriched in scaffolds seeded with DE cells lacking TP63 with the most significant related to liver, musculature, and musculoskeletal system, suggesting that DE differentiation is less restricted in the absence of TP63 (Supplementary Fig. 7a). Moreover, PE analysis identified 24 pathways of which the majority related to liver metabolism that were enriched at day 7 in scaffolds seeded with TP63-deficient versus wildtype DE cells. This suggests that TP63^−/−^ DE cells on the acellular lung scaffold might be permissive to differentiating into mesodermal or posterior foregut (hepatic) endoderm lineages rather than anterior foregut (lung) lineages only. Similar analysis at day 21 identified 68 anatomical terms in scaffolds seeded with DE cells lacking TP63. Some related to the liver but the majority associated with mesodermal-derived organ systems including muscular, skeletal, and circulatory (Supplementary Fig. 7a). GSEA showed that freshly sorted TRP63^−/−^ DE cells were enriched in genes related to mesenchyme development, formation, and differentiation (Fig. 6c and Supplementary Fig. 8a), confirming their potential to give rise to mesodermal-derived tissues on the scaffold. In addition, based on GSEA, endoderm cells lacking TP63 were more prone to epithelial mesenchymal transition (EMT) on the scaffolds than wildtype cells (Supplementary Fig. 8a, b). PE analyses revealed an overall enrichment in pathways related to liver metabolism (Supplementary Fig. 7a). Subsequent GSEA corroborated enrichment of various liver-associated metabolic pathways including that of triglycerides, fatty acids, and bile acids (Supplementary Fig. 7b). Together these RNA-seq results imply a role for TP63 in maintaining and restricting differentiation of definitive endoderm toward lung epithelial cells on lung acellular scaffolds.

## Discussion

Recellularization of whole-organ acellular scaffolds is an attractive approach for organ transplantation. Multipotent stem cells are an ideal cell source for reseeding acellular scaffolds, specifically those of complex multi-cellular organs like the lung. Various studies have shown that pre-differentiated lung progenitors under specific supplementation conditions can adhere, proliferate and differentiate to airway epithelium on acellular lung scaffolds^2,44,45^. We have shown that acellular lung ECM without supplementation promotes endoderm differentiation into proximal airway cells^8^. However, none of these studies have reported the temporal sequence of differentiation of endoderm toward airway cells on acellular lung scaffolds. Here, we show that TP63^+^ cells are the first cells to emerge on the acellular scaffolds, followed by TP63^+^/KRT5^+^/KRT14^+^ basal cells, and subsequently multiciliated and secretory epithelial cells. Removal of TP63 prohibited basal cell differentiation on the scaffolds and markedly impaired the formation of ciliated and secretory airway cells. These findings demonstrate that TP63^+^ progenitors are key for airway epithelial cell fate in the context of recellularization of a denuded lung with endoderm cells and highlight the regenerative potential of basal cells for tissue engineering of artificial airways.

Our temporal ultrastructural analysis of the scaffold cultures revealed the appearance of epithelial cells with primary cilia prior to epithelial cells containing bundles of motile cilia. A similar sequence of epithelial cilia appearance was noted during murine lung development, in agreement with previous studies^46,47^. Thus, differentiation of endoderm cells on the acellular scaffolds into proximal airway epithelial cells^8^ appears to replicate some features of upper airway epithelial development in situ where motile ciliated cells originate from primary ciliated cells^46,47^. Ultimately, endoderm cells seeded onto the acellular lung scaffolds differentiated into a pseudostratified epithelium resembling the adult upper respiratory tract of mice^48^. Ultrastructural recognizable basal cells emerged prior to ciliated and secretory epithelial cells, in line with them being putative progenitors^15^. Tracing the expression of TP63, a canonical basal cell marker^24,25,31^, corroborated the early appearance of TP63^+^ cells prior to any other cell types on the scaffold. During murine development, TP63^+^ cells appear in the primitive lung anlage at E9.5^26^. These multipotent cells are capable to give rise to proximal and alveolar epithelial lineages, although commitment to the alveolar lineage is lost at E10.5^26^. Other basal cell markers, including KRT5 and 14, have been reported to appear only after birth in vivo^24,25^. However, using lineage tracing in mice, TP63^+^ basal cells that later express KRT5 have been shown to arise around E13.5–14.5 in the developing lung^26^. In our decellularization-recellularization model, TP63 is the only basal cell marker to be expressed after 4 days of culture of definitive endoderm cells on the acellular scaffolds. It is plausible that these TP63^+^ cells represent the multipotent TP63^+^ cell population at the onset of lung development^26^. Previously, we have shown the presence of proximal SOX2^+^ and distal SOX9^+^ linage progenitors on the scaffold at day 7 of culture^8^, in line with TP63^+^ cells giving rise to both lineages in situ^26^. The distal SOX9^+^ progenitors were lost with prolonged culturing of the scaffolds^8^, in agreement with the lineage restriction of TP63^+^ cells to proximal cells seen during development^8^. SOX2 is expressed in the early lung endoderm^49^ and it directly regulates TP63 expression^50^. SOX2 expression increased rapidly on the scaffold^8^, suggesting that it may be responsible for the early emergence of the TP63^+^ basal cell lineage on the scaffold^50^. SOX2 expression in the developing airway epithelium is negatively controlled by WNT^51^. Whether WNT signaling is reduced in DE cells after seeding on acellular scaffolds is unknown. Previously, we have reported that molecules bound to heparan sulfate proteoglycans on the lung scaffold drive the differentiation of endoderm cells to proximal airway cells^8^. Additionally, interactions between integrin receptors on DE cells with scaffold extracellular matrix might change cell behavior and fate^52^. The contribution of these bound molecules and/or integrin adhesion complexes to basal cell fate remains to be investigated.

In the present study, TP63^+^/KRT5^+^ and TP63^+^/KRT14^+^ cells arise after 7 days of culture. In the adult lung, only a subset (~20%) of basal cells is positive for KRT14^27^, whereas in our decellularization-recellularization model the majority of basal cells expressing TP63 and KRT5 are also positive for KRT14. Both KRT14-negative and -positive subpopulations of basal cells in the adult lung upregulate their expression of KRT14 after injury^27,28^. In this context, all basal cells express KRT14 and these KRT14^+^ basal cells are direct progenitors for ciliated and club-like cells^28^. Our observation of most TP63^+^ basal cells being positive for KRT5 as well as KRT14 suggests that recellularization of an acellular scaffold with endoderm cells replicates the repair process of an airway after severe injury, i.e. when endoderm cells are seeded on an acellular lung scaffold, their differentiation directed by the scaffold follows a regenerative pathway instead of lung development. Acellular scaffolds simply resemble a denuded airway epithelium in wound repair^53,54^ to which progenitors attach and differentiate^55^.

TP63^+^/KRT5^+^ and TP63^+^/KRT14^+^ cells were absent when TP63-deficient endoderm cells were seeded onto acellular lung scaffolds, in agreement with the results seen in the epidermis and squamous epithelium of TP63 knockout mice, namely absence of KRT5- and KRT14-positive cells^18,56^. The differentiation to proximal airway epithelium on the scaffolds was also markedly altered in the absence of TP63. We observed a marked reduction in both multi-ciliated and secretory club cells contradictory to reports showing that absence of TP63 in mice does not prevent multi-ciliated and secretory cell formation^18,24,26^. One explanation for this discrepancy could be that we are only seeding endoderm cells on the scaffolds without any supplements and that important developmental growth factors and mesodermal components^57^ are missing to overcome the deletion of TP63. Loss of TP63 did, however, not prevent the expression of early lung and proximal lineage markers, NKX2-1 and SOX2, respectively. Alternatively, as suggested above, recellularization of an acellular scaffold seems to replicate airway regeneration, where in vivo, after injury, basal cells act as stem cells to regenerate the epithelium^15,35^. Since basal cells are not formed when acellular lung scaffolds are seeded with TP63-deficient DE cells, regeneration of the airway epithelium will not occur, which fits with the observed absence of multi-ciliated and club cells. The lack of TP63 could also be delaying differentiation and prolonged culturing could eventually result in differentiation of ciliated and secretory cells. Sustained gene expression of *Foxj1* and *Scgb1a1* in the TP63^−/−^ endoderm seeded scaffold cultures supports this idea; however, it can also be the results of a compensatory mechanism due to the lack of progenitors. Similar to findings in TP63 knockout animals^18,24,26^, we observed a limited pseudostratification of the epithelium lining the tubule-like structures of the scaffolds when seeded with TP63-deficient endoderm cells. This underscores the role of TP63 in epithelium stratification^58^.

The absence of TP63 did not increase the differentiation toward distal lung phenotypes. A few studies have shown a role for TP63^+^ cells in the regeneration of alveolar epithelium post-injury^59–61^. Specifically, a subpopulation of TP63^+^/KRT5^+^ epithelial cells in the intrapulmonary airways has been implicated to function as stem cells for the alveolar region after H1N1 influenza injury^62^. Cell fate tracing during influenza injury has suggested that these cells originate from TP63^+/^KRT5^−^ cells that acquire KRT5 expression after they migrate more distally^26^ and start to express alveolar cell markers^62^. Although numerous TP63^+^/KRT5^-^ and TP63^+^/KRT5^+^ cells emerged on the scaffolds when seeded with wildtype endoderm cells, differentiation to alveolar lineages did not occur, independent if the acellular lung scaffold was from the proximal or distal region^8^. Since recellularization of an acellular scaffold differs from influenza triggered structural repair, these findings suggest that the regenerative capabilities of TP63^+^ basal cells are heterogeneous and injury dependent. It is worthwhile to enforce that we are seeding the scaffolds solely with endoderm cells. The presence of mesenchymal cells might have triggered different responses as they have shown to promote differentiation of endoderm-derived lung progenitors to alveolar lineages^43,63^.

Although the absence of TP63 did not affect endoderm differentiation and growth, differential gene analysis immediately after definitive endoderm sorting revealed significant enrichment of genes related to mesenchyme development and epithelial to mesenchymal transition (EMT). EMT-associated genes were also increased in TP63^−/−^ endoderm seeded scaffolds after extended culture. These findings agree with TP63 maintaining epithelial integrity and limiting EMT^64–67^ and explains the enrichment in genes of mesodermal lineages even after initial selection for endoderm. RNA-seq further demonstrated that the differentiation occurring on the acellular scaffolds seeded with TP63-deficient endoderm cells diverges from those seeded with wildtype endoderm cells. Enrichment in liver anatomical terms and metabolic pathways suggests TP63-deficient cells on the scaffold may partially differentiate to hepatic lineages. Increased gene expression of *Albumin* and *FoxA3* (hepatic lineage markers) supports this shift in differentiation of TP63-deficient endoderm on acellular lung scaffolds toward posterior foregut endoderm. Although we observed an enrichment in genes associated with muscle, heart, and metabolic pathways, the number of mitochondria was less in tubule-lining epithelial cells on scaffolds seeded with TP63^−/−^ endoderm cells. Various studies have reported that per-cell mitochondria number varies between tissues and cell types, depending on metabolic need^68^. We speculate that the decrease in number of mitochondria is due to TP63^−/−^ endoderm seeded scaffolds lacking cells with motile cilia and, therefore, energy-consuming ciliary movements. Although mitochondria in TP63^−/−^ cells lining the tubule-like structures were larger in size, they had a similar shape as mitochondria of wildtype tubular-lining cells. Interestingly, we distinctly detected autophagolysosomes with organelle debris resembling mitochondria in the TP63^−/−^ tubular-lining cells, suggesting the occurrence of mitophagy. Whether the reduction in mitochondrial density in TP63^−/−^ lining cells is due to less mitochondrial biogenesis, increased mitophagy, and altered mitochondrial dynamics needs further investigation.

Summarizing, TP63 is needed to guide the differentiation of endoderm to basal cells and subsequently pseudostratified proximal airway epithelium on acellular lung scaffolds. Recellularization of a decellularized lung with endoderm cells appears to replicate airway epithelial wound repair and regeneration instead of lung development. Our findings support the use of pulmonary basal cells as a cell source for reseeding an acellular lung^45,69^. The recent finding that acellular lung scaffolds support region-specific epithelial differentiation of primary TP63^+^ basal cells better than artificial platforms like organoids or air-liquid-interface cultures^45^ emphasizes this concept.

## Methods

### Generation of TP63-deficient ES cells

To facilitate identification of a CRISPR introduced insertion/deletion, we designed a donor vector that contains a full transcriptional unit expressing a selection marker (puromycin) and a fluorescent protein (mCherry). PCR was used to amplify a 2.1 kb part of the pSicoR-Ef1a-mCh-Puro plasmid (a gift from Bruce Conklin, Addgene plasmid #31845; http://n2t.net/addgene:31845; RRID:Addgene_31845)^70^ encompassing the EF-1a promoter till the stop codon for puromycin. The primers were as follows: (forward primer) 5′-ggagcgcaccatcttcttcaaggcaagactcagacctcagtgaccccagcggccgcggatctgcgatcgctc-3′ and (reversed primer) 5′-ggagcgcaccatcttcttcaaggacgtcacatttcgta ttttatttaccgggcttgcgggtcatgcaccag-3′. The PCR generated DNA fragment was cloned into the pJET1.2/blunt cloning vector using the CloneJET PCR Cloning Kit (Thermo Fisher Scientific, #K1231). The cloned DNA sequence was verified by sequencing. In addition to the full transcriptional unit, the donor vector contained two guide RNA target sites at both ends. Once the donor vector is introduced into cells, a sgRNA targeting eGFP4, expressed by co-transfected lentiCRISPR-EGFP sgRNA 4 plasmid that contains inserts for Cas9, puromycin resistance, and EGFP sgRNA 4 (gift from Feng Zhang, Addgene plasmid #51763; http://n2t.net/addgene:51763; RRID:Addgene_51763)^71^, will cut these eGFP4 sites, thereby releasing a linearized full-length transcriptional unit. The linearized DNA can then be integrated into the genome after CRISPR introduced site-specific double DNA break and endogenous DNA repair mechanism. To achieve this, we performed DNA electroporation using the Neon™ Transfection System Kit (Thermo Fisher Scientific, #MPK10096). Briefly, mouse ES (129/Ola (Bry-GFP/Foxa2-hCD4) cells were harvested and resuspended in buffer (provided by Neon™ Transfection System Kit) to a final concentration of 5 × 10^7^ cells/ml. Three million cells in 120 μl resuspension buffer were mixed with 3 μg of donor vector, 1.3 μg of sgRNA vector for mouse TP63 (Applied Biological Materials, #K4404802), sequence: GCGCACTCACCCACATG) and 1.7 μg of lentiCRISPR-EGFP sgRNA 4. After electroporation using parameters optimized for the ES line, cells were seeded in a well of a 6-well plate, pre-coated with 0.1% gelatin in 2i pre-warmed growth media^72^. Media was changed one day after transfection and then every other day. Five days after transfection, 0.25 μg/ml puromycin was added to the media. One week later, single-cell clones resistant to puromycin and positive for mCherry were hand-picked and reseeded in 6-well plates (2 wells for every clone) pre-coated with 0.1% gelatin. After two weeks, genomic DNA was extracted from one of the wells with the individual clones and integration of donor sequence and mutation of the other allele were verified by DNA sequencing. After confirmation TP63^−/−^ ES cells were expanded and frozen in liquid nitrogen until use.

### ES cell differentiation and endoderm induction

Wildtype (129/Ola (Bry-GFP/Foxa2-hCD4; gift of Dr. G. Keller, University Health Network, Toronto) and TP63^−/−^ ES cells were maintained below passage 50 in the pluripotent state under feeder-free, serum-free culture in 2i media^72^. For spontaneous differentiation, single cells were cultured on non-treated culture plate (Sarstedt, #821473080) at a density of 50,000 cells/ml in DMEM (Gibco, #11995-065) supplemented with 10% FBS (Gibco, #10438-026) and for the first day 10 μM Y-27632 (Tocris,#1254) to allow for embryonic body (EB) formation. From day 3 till day 7, EBs were harvested every other day, pelleted, and resuspended in DMEM + 10% FBS. At day 7, EBs were collected and seeded into wells of a 12-well plate coated with 0.1% (w/v) gelatin at a density of 5-10 EB/well in DMEM + 10% FBS. The media was changed every other day for 14 days. At day 21, cells were fixed in 4% paraformaldehyde for 1 h and processed for expression of lineage markers of mesoderm (SMA), endoderm (FOXA2), and ectoderm (TUBB3) using immunofluorescence. For endoderm induction, single cells were cultured on non-treated culture plates (Sarstedt, #821473080) at a density of 50,000 cells/ml in serum-free differentiation media (SFDM)^73^ to allow for EB formation. After 2.5 days, EB were harvested, washed, and cultured in SFDM supplemented with 50 ng/ml Activin A (StemCell Technologies, #78132) for an additional 2.5 days. At day 5, EBs were harvested, dissociated into single cells with TryplE (Gibco, #12605028), and labeled with CD117 (c-KIT) and CD184 (CXCR4) antibodies. Double positive (c-KIT^+^/CXCR4^+^) definitive endoderm (DE) cells were sorted using MoFlo Astrios (Beckman Coulter) and data analysis was carried out using Kaluza (Beckman Coulter).

### Rat Lung decellularization

All animal experimentations were approved and carried out in accordance with the animal care committee of the Hospital for Sick Children Research Institute (protocol #1000043112). Previously, we reported that there are no intra- and inter-species differences between mouse and rat cell-scaffold combinations^74^. Due to the rat lung being larger, it provides us with more scaffolds per lung, thereby reducing biological variation. The decellularization procedure of rat lung has been described previously^74^. Briefly, lungs were perfused with 10 U/mL heparinized HBSS^-^ (Millipore Sigma, #H0777) to remove blood cells. Lungs were then decellularized by sequential tracheal lavages with 8 mM CHAPS, 25 mM EDTA, 1 M NaCl in PBS, followed by extensive rinsing with PBS. Thick (400 µm) vibratome sections (Leica Microsystems GmbH) of the decellularized lung were incubated in 90 U/mL benzonase (Millipore Sigma, #70664-3) for 24 h and then treated with 200 U/mL penicillin/streptomycin and 25 µg/mL amphotericin B (Gibco, #15290-018) for another 24 h. The decellularized scaffolds were kept in the antibiotic solution at 4 °C until the day of recellularization.

### Reseeding acellular rat lung scaffolds

Decellularized lung scaffold sections were washed with PBS and conditioned in SFDM prior to recellularization. The 400 µm acellular scaffolds were transferred on Whatman™ 8 μm Nuclepore™ hydrophobic floating membranes (Millipore Sigma, # 110614) and seeded with c-KIT^+^/CXCR4^+^ sorted DE cells (100,000 cells/scaffold). SFDM media was changed every other day for up to 21 days.

### Attachment and proliferation analysis

After sorting, wildtype and TP63^−/−^ (c-KIT^+^/CXCR4^+^) definitive endoderm cells were plated (300,000 cells/well) on 0.1% (w/v) gelatin-coated 6-well culture plates in SFDM. Media was changed every other day and images were taken 24 h and 72 h after seeding using a Leica DEMIL LED microscope with a Leica MC170 HD camera.

### Electron microscopy (EM) analysis

Cell-scaffold cultures were collected at various intervals and fixed in 2.5% glutaraldehyde in 0.1 M sodium cacodylate buffer, pH7.4. For routine transmission EM samples were post-fixed in 1% osmium tetroxide, dehydrated in an ascending series of ethanol, infiltrated with propylene oxide, and embedded in Quetol-Spurr resin. Ultrathin sections were then cut with a Leica EM UC7 ultramicrotome, mounted on grids, and stained with uranyl acetate and lead citrate prior to image acquisition with a FEI Tecnai 20 TEM. For scanning EM samples were post-fixed in 1% osmium tetroxide, dehydrated in an ascending series of ethanol, critical point dried using a Bal-tec CPD030 critical point dryer, and mounted on aluminum stubs. Samples were gold-coated using a Leica ACE200 sputter coater and examined and photographed with a FEI XL30 SEM. For immunogold EM samples were processed as described previously^75^. Ultrathin sections were cut to gold thickness and placed onto 400-mesh copper grids for immunogold. Immunogold labeling was performed as previously^75^ using 1:200 diluted rabbit anti-TP63 (Cell Signaling) or anti-KRT5 (Abcam) antibodies followed by a 1:300 diluted 10-nm gold-conjugated goat anti-rabbit IgG (Nanoprobes). Samples were then stained with 3% (w/v) uranyl acetate and 1% (w/v) lead citrate and examined on a Philips 430 electron microscope.

### Histological and immunofluorescence analysis

Cell-scaffold cultures were collected at various durations of culture and fixed in 4% paraformaldehyde (PFA), dehydrated in an ascending series of ethanol, transferred to xylene prior to embedding in paraffin and sectioned. Thin 5 μm sections were rehydrated and used for either hematoxylin & eosin or immunofluorescence staining. For immunofluoresence (IF), sections were rehydrated and heat-induced antigen retrieval with 10 mM citrate buffer, pH 6.0 was performed. Sections were blocked with 10% normal donkey serum/1% BSA/0.1% Tween-20 in PBS for 1 h at room temperature. Slides were then incubated with primary antibodies in blocking solution at 4 °C overnight in a humidified chamber, rinsed with PBS, and then incubated with fluorescent-conjugated secondary antibodies diluted in blocking solution for 1 h at room temperature. Nuclei were counterstained using DAPI (Thermo Fisher Scientific, #D1306). Details of antibodies are provided in Supplementary Table 1. Images were captured with Leica CTRMIC 6000 confocal microscope and Hamamatsu C910013 spinning disc camera (Leica Microsystems GmbH). The sensitivity and intensity were unchanged during image acquisition within the same experiment and images were analyzed with Volocity software (Perkin Elmer). For immunostaining quantification, at least 10 images of tubule-like epithelial structures per scaffold were taken at ×40. Immuno-positive and total number of cells (DAPI positive) in each structure were counted. For measurements of circumference of tubule-like structures, H&E images were taken at ×5 with a Leica DM6000 microscope fitted with a Hamamatsu ORCA-ER B/W CCD camera and analyzed with Image J software. All tubule-like structures in the images with a visible lumen were measured.

### Real-time qPCR analysis

RNA was extracted from cell-scaffold cultures using PureLink RNA micro scale kit (Thermo Fisher Scientific, # 12183016) and cDNA synthesis was carried out with 0.1–1 μg of RNA using SuperScript^TM^ IV VILO^TM^ Master Mix (Invitrogen # 11766050), according to the manufacturer’s protocol. Twenty ng of template cDNA was used for real-time PCR (40 amplification cycles) with SYBRSelect Master Mix (Applied Biosystems, 34472908) using murine specific primer sets, listed in Supplementary Table 1. Analysis was performed using StepOnePlus qPCR (Applied Biosystems). Gene expression was normalized to RNA polymerase II and expressed relative to selected appropriate positive or negative controls.

### RNA sequencing

Wildtype definitive endoderm (DE) cells, TP63-deficient DE cells, and scaffolds (3-4 scaffolds/experiment, ≥3 separate experiments) seeded with wildtype or TP63-deficient DE cells were collected after 7 or 21 days of culture and placed in lysis buffer provided with PureLink RNA micro scale kit (Thermo Fisher Scientific, #12183016). The cell-scaffold samples were homogenized by sonication and RNA was extracted following the instruction of the manufacturer. RNA was sent to The Centre for Applied Genomics (TCAG, The Hospital of Sick Children Research Institute) for purity assessment and RNA library construction using NEBNext Ultra II RNA Library Prep Kit (New England Biolabs). Libraries were sequenced on an Illumina Hiseq 2500 using paired-end mode (2 × 126). The raw base call files were converted to fastq files using bcl2fastq2 v2.20. Alignment was perform using STAR software and the reference genome Mus musculus mm10 (GRCm38.p6). Differential analysis was executed with DESeq2. The ranked differentially expressed genes with a FDR of less than 0.05 were selected for further analysis with Mouse mine (MouseMine.org) where a FDR of less than 0.05 with a correction test (Benjamini Hochberg procedure) was used to visualise pathway enrichment and enriched anatomy (EMAPA) terms in selected differentially expressed genes. Finally, gene set enrichment analysis (GSEA) was performed using Mouse_GOBP_ALLpathway_no_GO_iea_march_01_2019. Enrichment analysis was performed using the GSEA Preranked module using the list of all significantly altered genes (FDR < 0.05), ranked by the fold change expression with recommended default settings. Gene sets with an FDR < 0.05 were considered significantly enriched.

### Statistical analysis

All data are expressed as mean ± SEM. Statistical analysis was performed using GraphPad Prism 7.01 software. Comparison of data between two groups was done using paired Student t-test. Significance was denoted as *p < 0.05.

## Supplementary information

Supplemental Material

## Data Availability

Sequence data that support the findings of this study have been deposited in the NCBI Sequence Read Archive with the accession code PRJNA682756 (http://www.ncbi.nlm.nih.gov/bioproject/682756). Non sequencing data and materials generated for the study are available from the corresponding author on reasonable request.
