# Supplementary material for: TP63 basal cells are indispensable during endoderm differentiation into proximal airway cells on acellular lung scaffolds
Source: NPJ Regen Med. 2021 Mar 5;6:12. doi: 10.1038/s41536-021-00124-4 (PMC7935966; doi:10.1038/s41536-021-00124-4)
Supplement: Supplementary file 1 — Supplemental Material [file 41536_2021_124_MOESM1_ESM.pdf]

TP63 Basal Cells Are Indispensable during Endoderm Differentiation into Proximal Airway Cells on Acellular Lung Scaffolds

Claudia Bilodeau, Sheri Shojaie, Olivia Goltsis, Jinxia Wang, Daochun Luo, Cameron Ackerley, Ian Rogers, Brian Cox, Martin Post

Supplementary Figures and Tables

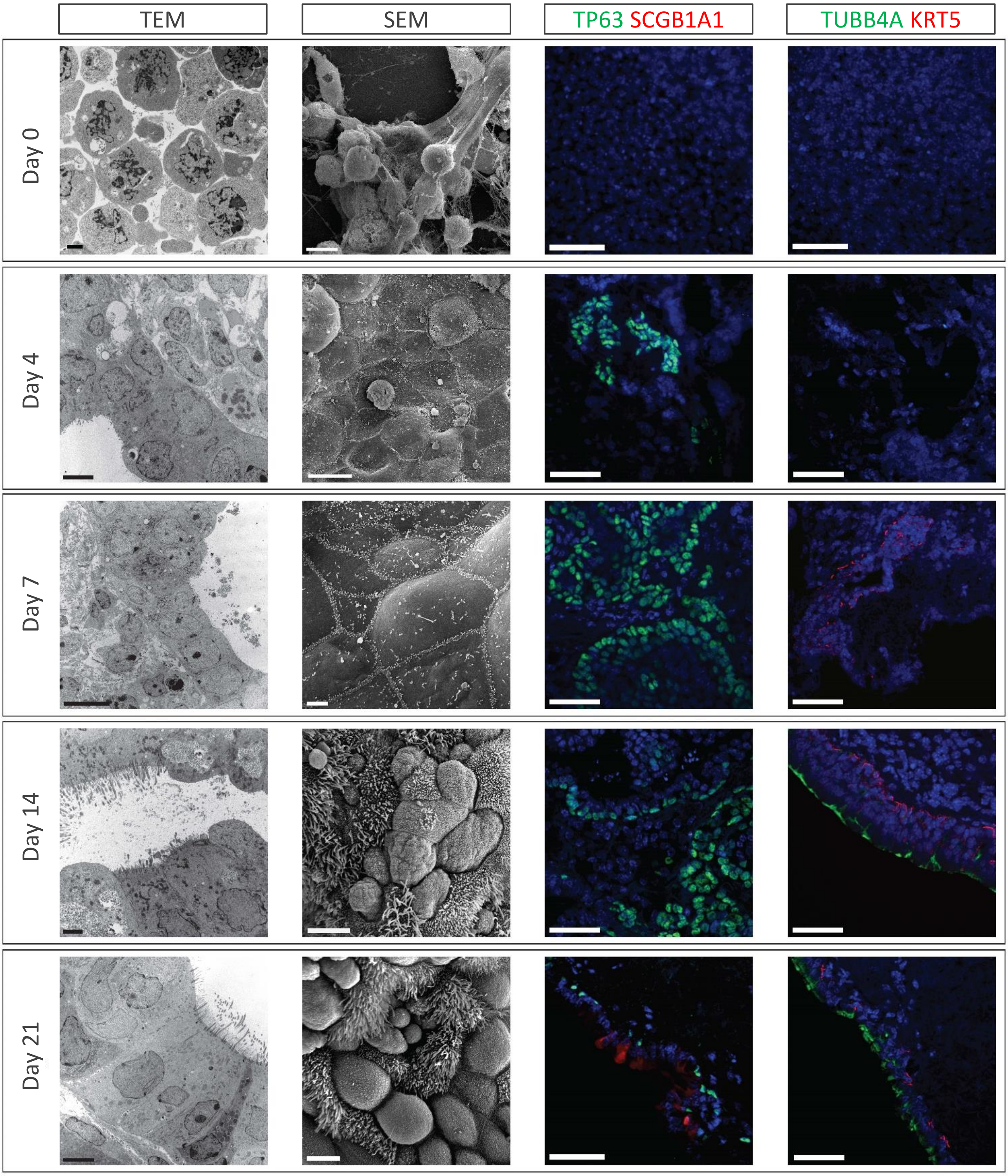

Supplementary Figure 1. Progression of differentiation of definitive endoderm (DE) cells to airway cells on decellularized lung scaffolds. (**Left 2 panels**) Representative TEM and SEM images of differentiation of DE cells into airway cells on acellular lung scaffolds during 0-21 days of culture. (**Right 2 panels**) Representative immunofluorescence confocal images of DE cells differentiating into airway cells during 0-21 days of culture on acellular lung scaffolds. Sections were stained for basal (TP63-green; KRT5-red), club (SCGB1A1-red) and ciliated (TUBB4A-green) cells. Organization and early differentiation of DE cells into TP63^{+} basal cells are already evident after 4 days of culture. Ciliated airway cells are detected at day 14 of culture while club cells appear at day 21. SEM, scanning electron microscopy; TEM, transmission electron microscopy. Scale bars: TEM 2µm, SEM 10µm, IF 25µm.

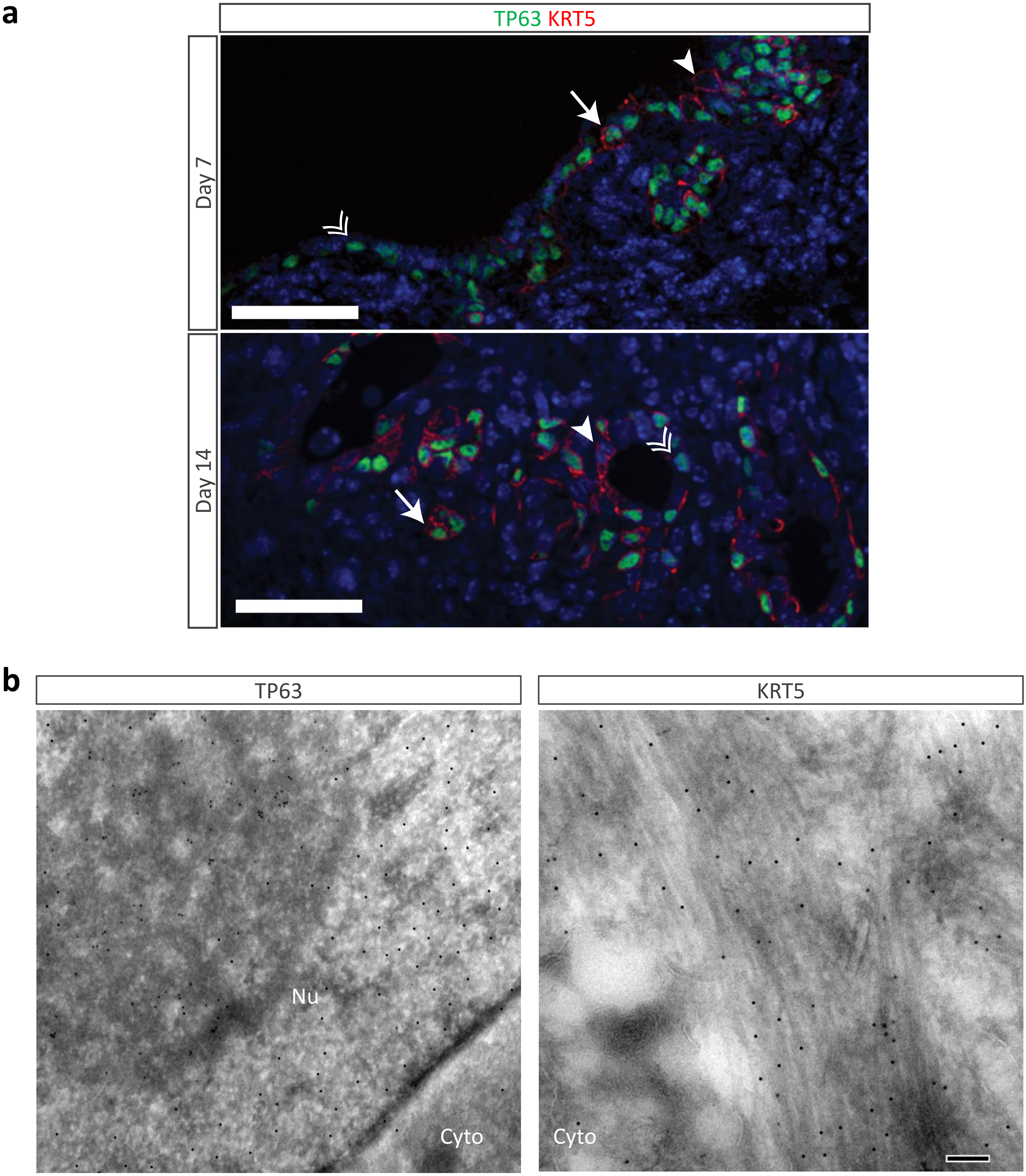

Supplementary Figure 2. (a) Immunofluorescence confocal images of DE cells on acellular lung scaffolds after 7 and 14 days of culture co-stained for TP63 and KRT5. At both days, many cells are double positive for both basal cell markers (arrow), but there are a few cells that express only TP63 (double arrow head) or KRT5 (arrowhead). Scale bar: 25μm. **(b) Immunogold labelling of a basal cell for TP63 and KRT5 at day 7 of culture of endodermal cells on decellularized lung scaffolds.** TP63 localizes to the nucleus and KRT5 is present in bundles that form the intermediate filaments within the cell. Nu, nucleus; Cyto, cytosol. Scale bar: 500 nm.

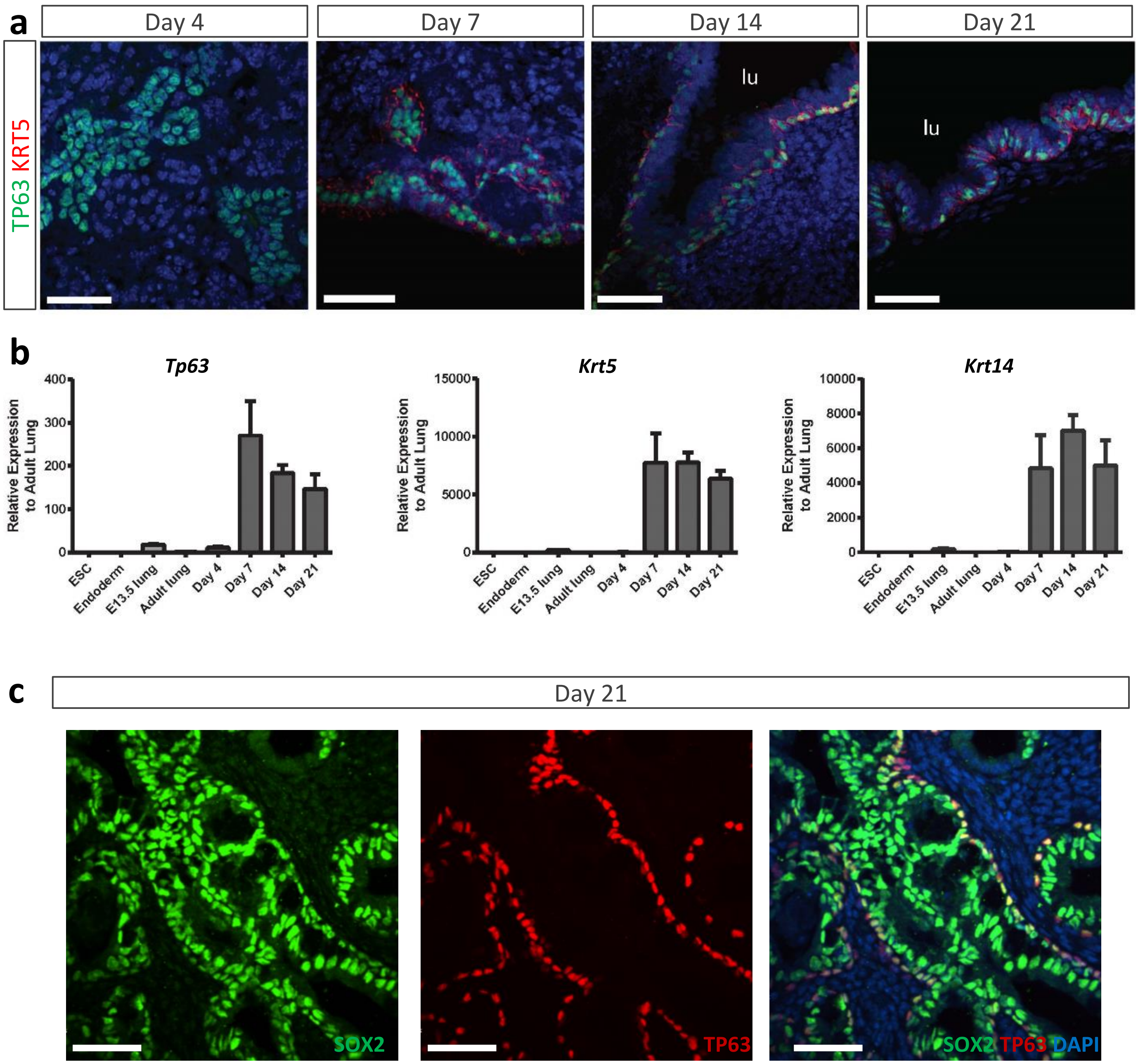

Supplementary Figure 3. Temporal expression of basal cell lineage markers in decellularized lung scaffolds seeded with definitive endoderm (DE) cells. (**a**) Representative immunofluorescence confocal staining for TP63 and KRT5 of DE cells seeded on acellular lung scaffolds after various times of culture. With longer duration of culture spatial expression of double positive TP63/KRT5 basal cells starts to resemble that of native mature airways. Scale bar: 25 µm. (**b**) Real-time PCR analysis of *Tp63* (adapted from previously published data Shojaie et al. 2015), *Krt5* and *Krt14* gene expression in cultures of acellular lung scaffolds seeded with DE cells. Mean ± SEM, n=3 separate DE-scaffold cultures. (**c**) Representative immunofluorescence confocal staining for SOX2 and TP63 of cells on acellular lung scaffolds, 21 days after seeding with DE cells. Cells lining the tubule-like structures are SOX2 positive while underlying basal cells stain positive for SOX2 and TP63. Scale bar: 50 µm

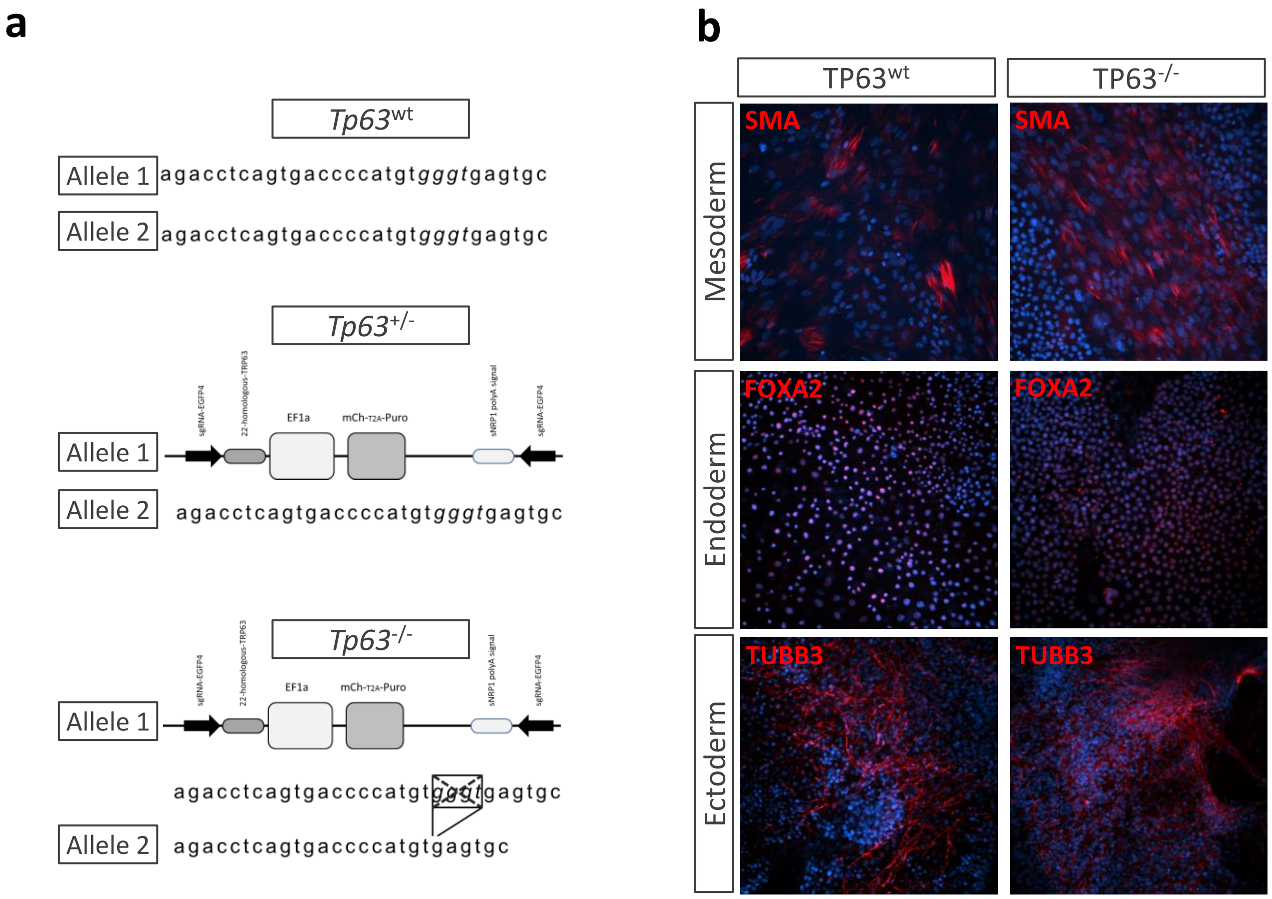

Supplementary Figure 4. TP63-deficient embryonic stem cells created by CRISPR/Cas9 differentiate into the three germ layers. (**a**) CRISPR/Cas9 strategy used to generate TP63- deficient ($TP63^{-/-}$) ES cells. (**b**) Immunofluorescence confocal staining for alpha-smooth muscle actin (SMA), forkhead box protein A2 (FOXA2) and tubulin beta-3 chain (TUBB3) of embryonic bodies cultured for 21 days on gelatin-coated plates in DMEM+10% FBS. Positive staining demonstrates that loss of TP63 did not affect the undirected differentiation capacity of the ES cells into SMA^{+} mesoderm, $FOXA2^{+}$ endoderm and $TUBB3^{+}$ ectoderm.

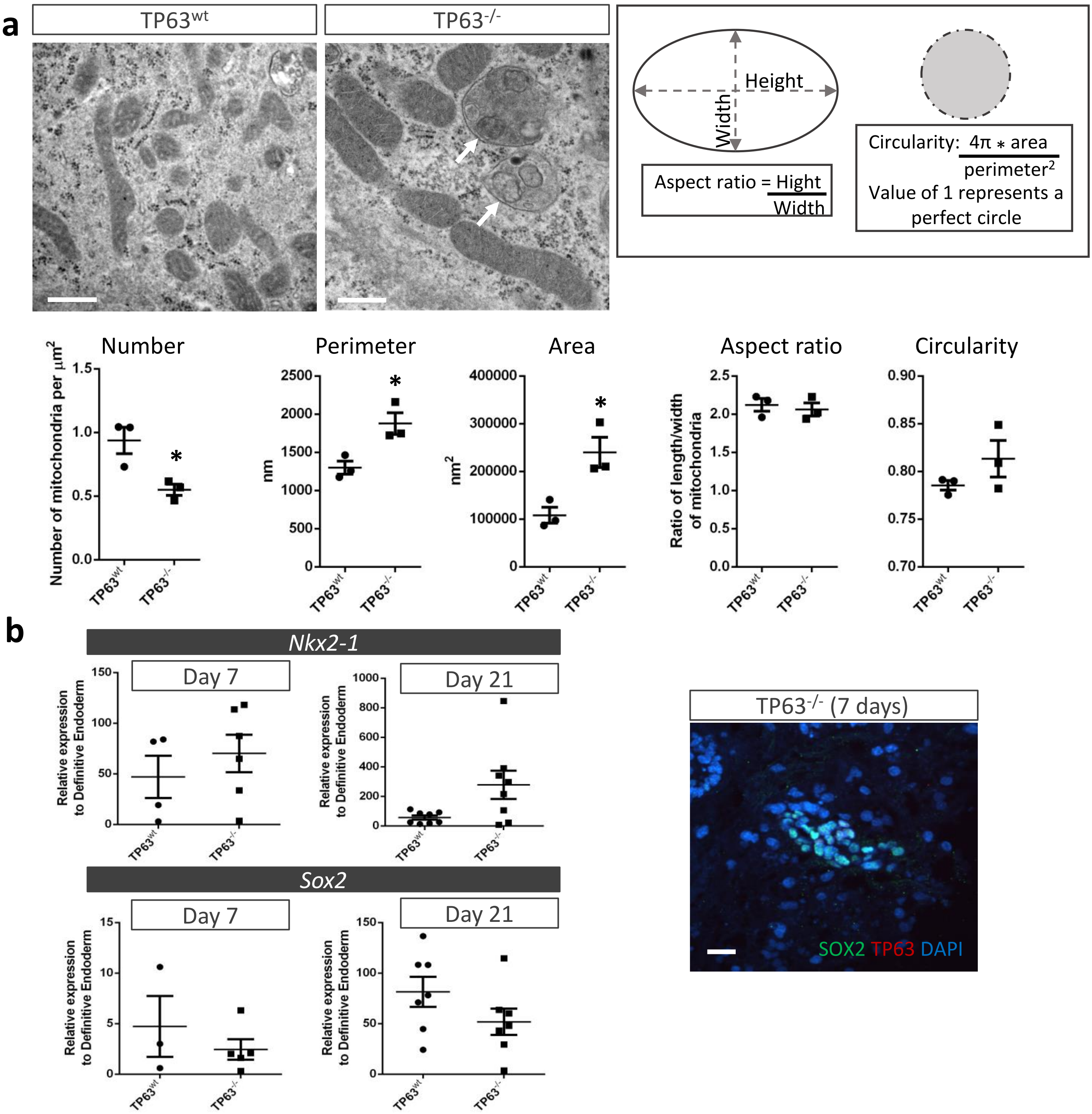

Supplementary Figure 5. (a) Epithelial cells lining the tubule-like structures on acellular lung scaffolds seeded with TP63-deficient DE cells have less but larger mitochondria without any change in shape compared to epithelial cells on scaffolds seeded with wildtype DE cells. Top: Representative TEM images of mitochondria within epithelial cells lining the tubule-like structures, scale bar: 500nm. Note presence of autophagolysosomes (arrows) containing organelle fragments in TP63-deficient DE cultures. Bottom: Measurement of mitochondrial parameters (number, surface area, perimeter, aspect ratio, and circularity) were obtained from TEM images (n= 3 separate scaffolds; ≥ 16 images/scaffold, *p<0.05). **(b) Loss of TP63 does not affect expression of lung progenitor (NKX2-1) and proximal lung lineage (SOX2) markers.** (Left panels) Gene expression of *Nkx2-1* and *Sox2* at day 7 (n= 3-5 separate scaffold cultures) and day 21 (n= 7-8 separate scaffold cultures) was assessed by quantitative PCR. (Right panel) Immunofluorescent confocal imaging confirms that after 7 days of culture clusters of cells on scaffolds seeded with TP63-deficient cells are positive for proximal lineage marker SOX2. Scale bar: 25 µm.

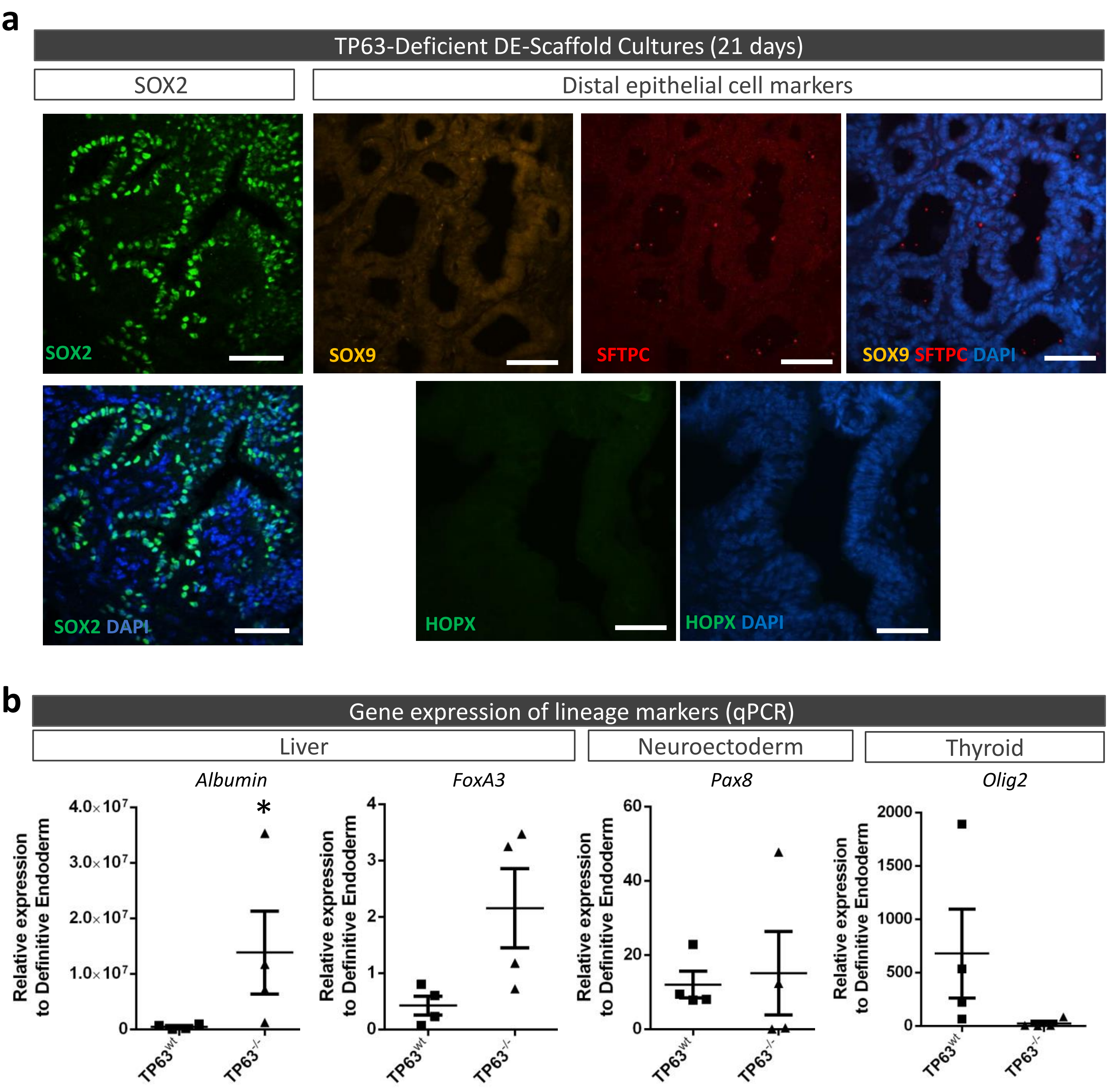

Supplementary Figure 6. (a) TP63-deficient definitive endoderm (DE) cells do not differentiate into distal alveolar cells after 21 days of culture on decellularized lung scaffolds. Immunofluorescent confocal images show that cells lining the tubule-like structures are positive for proximal lineage marker SOX2 but negative for distal lineage marker SOX9, alveolar progenitor marker HOPX, and alveolar type II cell marker SFTPC. Scale bar: 50 µm. **(b) TP63-deficient DE cells do differentiate into hepatic, but not thyroid, forebrain, lineages when cultured for 21 days on decellularized lung scaffolds.** Gene expression of lineage markers for liver are upregulated in acellular lung scaffolds seeded with $TP63^{-/-}$ DE cells, whereas those for thyroid and neuroectoderm were not significantly between acellular lung scaffolds seeded with either wildtype or $TP63^{-/-}$ DE cells (n= 5 separate scaffold cultures, *$p<0.05$).

a

EMAPA term significantly enriched after 7 days on acellular scaffold	EMAPA term significantly enriched after 21 days on acellular scaffold	Pathways significantly enriched after 7 days on acellular scaffold
Increase in TP63$^{-/-}$ (59 terms)	Increase in TP63$^{-/-}$ (68 terms)	Increase in TP63$^{-/-}$ (25 pathways)
liver lobe	musculoskeletal system	Metabolism
liver	liver and biliary system	Regulation of IGF transport and uptake by IGFBPs
liver and biliary system	connective tissue	Metabolism of lipids
visceral organ system	cardiovascular system	Cholesterol biosynthesis
musculoskeletal system	bone	linoleic acid metabolism
alimentary system	skeleton	Ethanol oxidation
gut	musculature	Metabolism of steroids
Musculature	heart	Fatty acid metabolism
skeletal system	skeletal muscle	Triglyceride metabolism
foregut-midgut junction epithelium	skeletal musculature	Plasma lipoprotein assembly, remodeling, and clearance

b

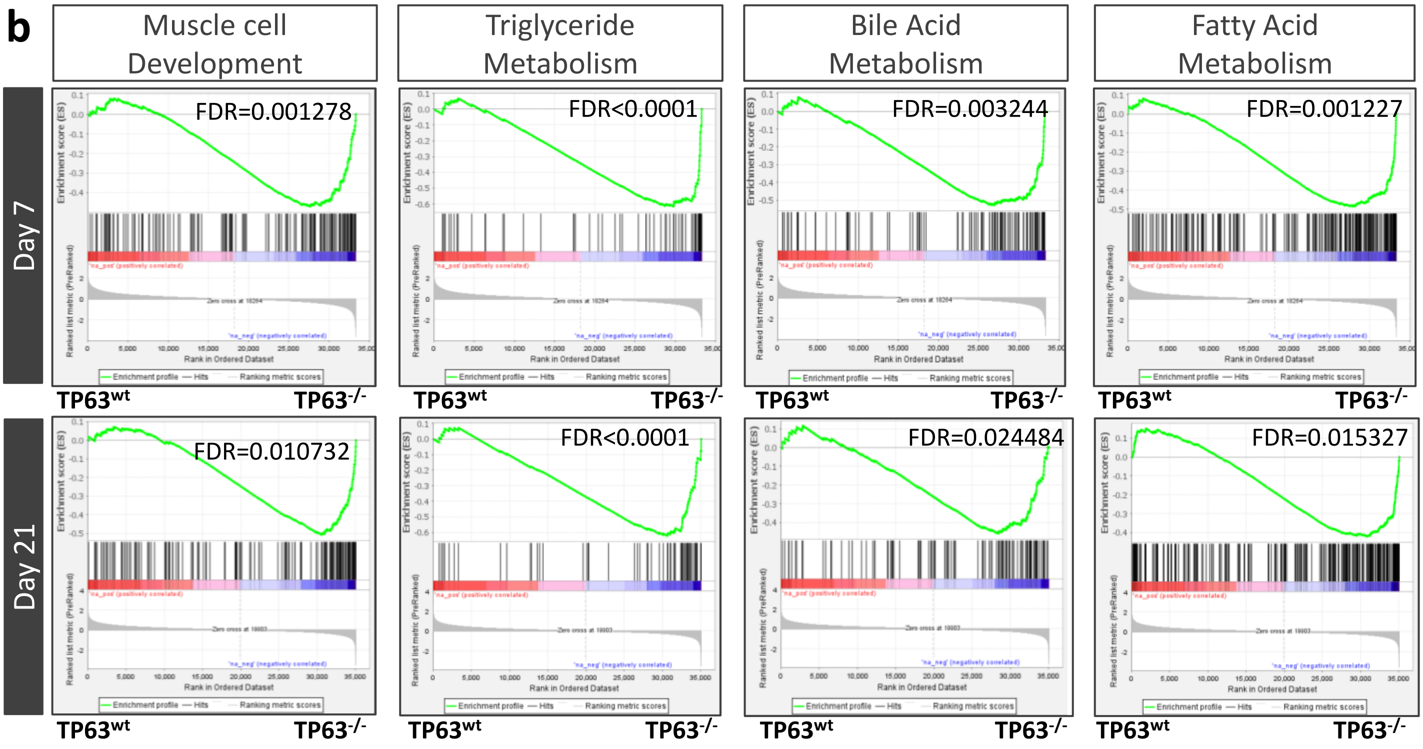

Supplementary Figure 7. Transcriptome analysis demonstrates increased expression of genes related to non-lung organs in TP63$^{-/-}$ DE cell-scaffold cultures. (**a**) Ten highly significant anatomical terms (EMAPA) and pathways enriched in TP63$^{-/-}$ cells seeded on acellular lung scaffolds. The RNA-seq analysis reveal an increase in non-lung gene expression at 7 (**a**) and 21 days of culture compared to wildtype DE cell-scaffolds. (**b**) GSEA of genes associated with muscle development and various liver metabolic pathways after 7 and 21 days of culture of TP63^{wt} and TP63$^{-/-}$ DE cells on acellular scaffolds. Analysis was performed on differentially expressed genes with a FDR smaller than 0.05. RNA isolation and sequencing at day 7 and 21 were repeated ≥ 3 times using 4 separate wildtype and 3 separate TP63-deficient DE cell-scaffold cultures, respectively.

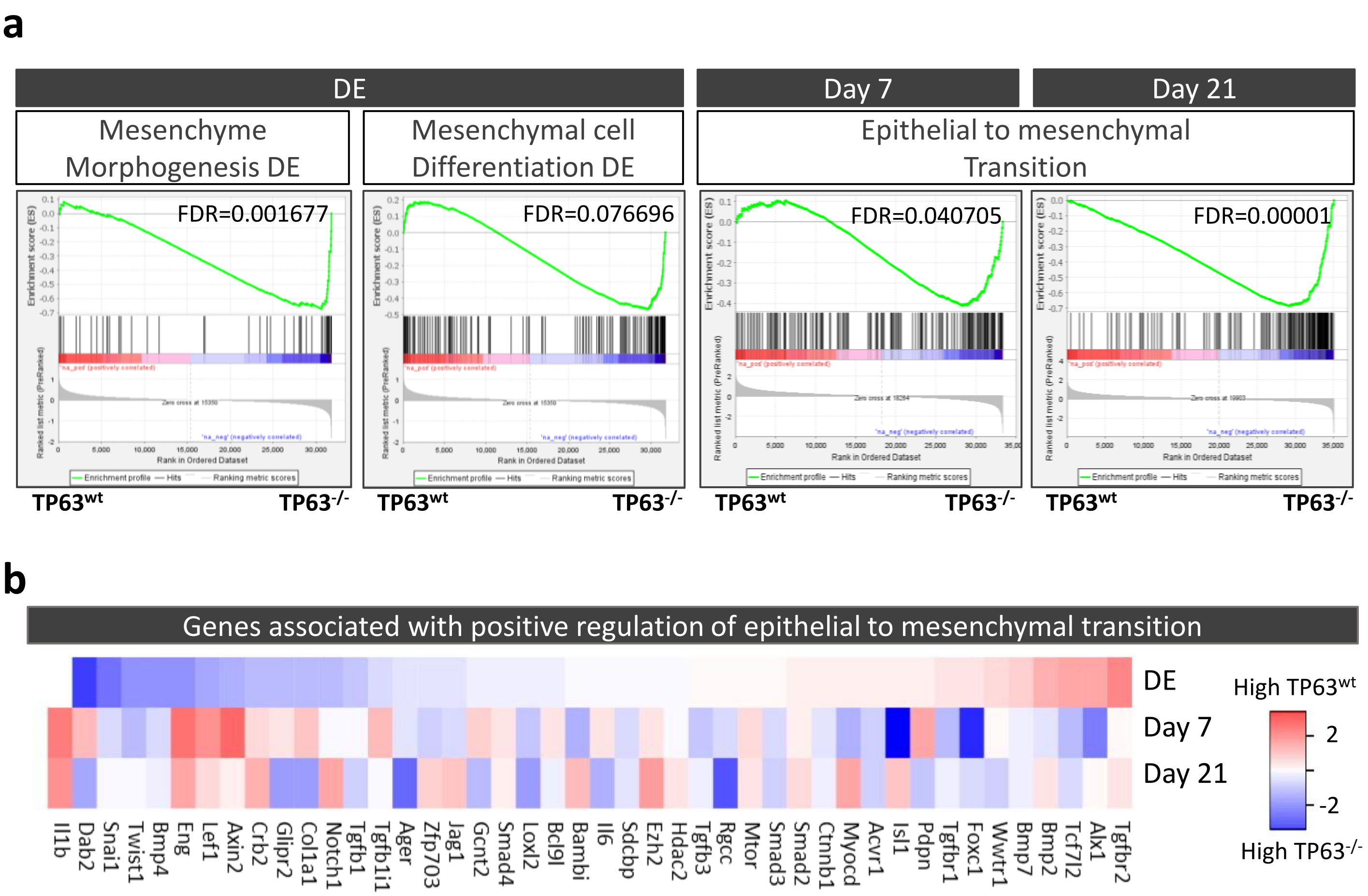

Supplementary Figure 8. **TP63-deficient DE cells seeded on decellularized lung scaffolds undergo epithelial mesenchymal transition**. (**a**) GSEA reveals that TP63$^{-/-}$ DE cells are enriched in genes associated with mesenchyme development when compared to wildtype DE cells. After 7-21 days of culture, TP63$^{-/-}$ cells seeded on the acellular scaffolds maintain enriched genes associated with epithelial-mesenchymal transition. (**b**) Heatmap demonstrates a trend in enrichment in genes positively associated with epithelial to mesenchymal transition in TP63$^{-/-}$ cells cultured on acellular scaffolds. RNA isolation and sequencing were repeated ≥3 times using 3 separate wildtype and TP63-deficient DE cell preparations, and 4 separate wildtype and 3 separate TP63-deficient DE cell-scaffold cultures for day 7 and 21.

Supplementary Table 1. Information regarding antibodies and qPCR primers

Antibodies

Target	Host, Conjugation	Company	Catalogue number	Application, Dilution
**Primary Antibodies**				
c-KIT	Rat, PE-CY7	BD Biosciences	558163	FACS, 1:100
CXCR4	Rat, APC	BD Biosciences	558644	FACS, 1:100
KRT5	Rabbit, non-conjugated	Abcam	Ab24647	IF, 1:500
KRT14	Mouse, non-conjugated	Lab vision	MS-115-PABX	IF, 1:500
NGFR	Rabbit, non-conjugated	Abcam	Ab8874	IF, 1:500
PDPN	Rabbit, non-conjugated	Abcam	Ab109059	IF, 1:200
SCGB1A1	Goat, non-conjugated	Santa Cruz	Sc-9772	IF, 1:600
TP63	Mouse, non-conjugated	Santa Cruz	Sc-8431	IF, 1:200
TUBB4A	Mouse, non-conjugated	Biogenex	MU178-uc	IF, 1:500
TP63	Rabbit, non-conjugated	Cell signaling	56687S	IF, 1:100
SPC	Rabbit, non-conjugated	Abcam	AB40879	IF, 1:500
SOX9	Goat, non-conjugated	R&D systems	AF3075	IF, 1:400
HOPX	Mouse, non-conjugated	Santa Cruz	Sc-398703	IF, 1:50
CDH1	Mouse, non-conjugated	BD Biosciences	610181	IF, 1:50
SMA	Mouse, non-conjugated	Dako	M0851	IF, 1:100
FOXA2	Goat, non-conjugated	Santa Cruz	Sc-6554	IF, 1:100
TUBB3	Mouse, non-conjugated	Millipore	MAB1637	IF, 1:100
SOX2	Goat, non-conjugated	R&D systems	AF2018	IF, 1:500
**Secondary Antibodies**				
Goat IgG	Donkey, Alexa Fluor 546	Invitrogen	A11056	IF, 1:200
Mouse IgG	Donkey, Alexa Fluor 488	Invitrogen	A11055	IF, 1:200
Rabbit IgG	Donkey, Alexa Fluor 647	Invitrogen	A31573	IF, 1:200
Rabbit IgG	Goat, 10nm colloidal gold	Nanoprobes	GA1013	Gold labeling, 1:300

FACS: Fluorescence-activated cell sorting; IF: Immunofluorescence staining

qPCR primers

Gene	Primer Source, Catalogue number/sequence	
Albumin	CCTAGGAAGAGTGGGCACCAAGTGT	AGCAGAGAAGCATGGCCGCCTTTC
Foxa3	Qiagen, QT01657705	
Foxj1	Qiagen, QT00111097	
Krt5	Qiagen, QT02262169	
Krt14	Qiagen, QT00114422	
Olig2	AATGCGCGATGCGAAGCTCTTT	AAGCCCACGTTGTAATGCAGGT
Pax8	TCGACTCACAGAGCAGCAGCAGT	AGGTTGCGTCCCAGAGGTGTATT
Sox2	Qiagen QT01539055	
Scgb1a1	Qiagen, QT00105266	
Tg	Qiagen, QT00116592	
Trp63	Qiagen, QT00197904	
Nkx2-1	TATGCTTCCATGGCCCTGAACT	TTTCCTATCTCCAGCGTCTGTCCT

Supplementary Table 2. Differentially expressed genes between TP63$^{-/-}$ and wildtype cells seeded and cultured on decellularized lung scaffolds after 7 and 21 days of culture. RNA isolation and sequencing were repeated ≥ 3 times using 4 separate wildtype and 3 separate TP63-deficient DE cell-scaffold cultures. The 100 most significant differentially expressed genes (FDR<0.0005) at day 7 and 21 of culture are shown.

Top 100 differentially expressed genes							
Day 7				Day 21			
Acss2	Tst	Acsl1	Slc6a13	Zzz3	Zdhhc8	Srek1	Flot1
Gstm7	Hgfac	Slc4a11	Btbd19	H2-Q7	Matn2	Htr2a	Reg4
Elovl2	Syde2	Necab1	Cited1	Ptx3	Fam131a	Tango2	Hs6st2
Isl1	Chst7	Med12l	Dgat2	Hamp	Bclaf1	Shh	Gbp5
Eva1a	Foxf2	Fgg	Hey1	Sftpb	Cpxm1	Clca3a2	Clca2
Gng5	Aldh1b1	St6gal1	Cldn14	Dgcr14	Bfsp1	Heatr3	Bcl10
Mmd2	C1s1	Apof	Abi3bp	Usp33	Ube2d3	Ppm1g	Plekha4
Adra2c	Gria2	Rabggtb	Gja3	Folh1	Zranb2	Naalad2	Dusp15
Doc2b	Gstm5	Stc1	Ccser1	Pdia5	Parn	Anks3	Rgs2
Scd2	Znhit6	Hadh	Ocstamp	Plin4	Dexi	Ifi44l	Abcb7
Serpinf1	Zzz3	H2-K1		Carhsp1	Olfm2	Tpr	
Srsf11	Synpo2	Ntrk3		Ltc4s	Mybpc2	Tyro3	
Nfe2	Col9a1	Scn3a		Srsf11	Lctl	Art4	
Tox2	Sfn	Itih3		Sh3glb1	Hs2st1	P3h1	
Zranb2	Pnpla3	Prox1		Metap1	Mapk1	Apol6	
Arhgap30	Prodh2	Litaf		Adgrl2	Scarf2	Bco1	
Serpinc1	Hfe2	Tmem88		Stap1	Creb5	Naa60	
Matn2	Pklr	Dgcr14		Cd40	Pitx1	Vasn	
Onecut3	Nkx2-1	Gas2		Gdf15	Galns	Serpinh1	
Creb3l3	Fpgt	Gna15		Znhit6	Pkn2	Socs2	
Fam73a	Slc38a3	Usp33		Dvl3	Csf2rb2	Arsa	
Ankrd13c	Adamts18	Gstt1		Rpf1	Gapvd1	Zfp263	
Lss	Gpx2	Ehhadh		Ppp1r3c	Arvcf	Vps8	
Bcl10	Paqr9	Adh1		Ppil2	Cryz	Mettl22	
Tcf24	Lrrc40	Ube2d3		Tspan5	Slc25a25	Man2a2	
Foxe3	Ush1g	Fads2		Hand2	Slc36a2	Nudt16l1	
Depdc1a	F7	Cpne4		Abcc5	Gpx3	Slc43a2	
Itih5	Hoga1	Cadm4		Mycl	Pah	Ppp3ca	
Slc36a2	Igfbp1	Glyctk		Slc9a9	Ssx2ip	Ccl6	
H2-Q10	Mgam	Sh3gl2		Ankrd13c	Bfar	Lztr1	
